# The relationship between childhood interpersonal and non-interpersonal trauma and autobiographical memory: a systematic review

**DOI:** 10.3389/fpsyg.2024.1328835

**Published:** 2024-01-17

**Authors:** Giovanni Borrelli, Annachiara Lamberti Zanardi, Claudia Scognamiglio, Vincenza Cinquegrana, Raffaella Perrella

**Affiliations:** ^1^Department of Human Sciences, Guglielmo Marconi University, Rome, Italy; ^2^Department of Psychology, University of Study of Campania “Luigi Vanvitelli”, Caserta, Italy

**Keywords:** childhood maltreatment, overgeneral memory, impairment of cognitive function, earliest memories, adverse childhood experience

## Abstract

Childhood trauma can have negative effects on several domains of mental functioning, including Autobiographical Memory (AM). Conflicting results emerge in the scientific literature regarding the effects of childhood trauma on AM. In this review, we explored the relationship between the childhood trauma and AM, classifying childhood trauma as interpersonal, non-interpersonal and overall (interpersonal and non-interpersonal). We carried out a systematic literature review, following the guidelines of the Preferred Reporting Items for Systematic Reviews and Meta-analyses (PRISMA statement). From searching the PubMed, Scopus, and Web of Science databases, we identified 48 studies conducted from 2014 to 2023, which were included when they: (a) were written in English, (b) investigated the relationship between AM and childhood trauma, (c) included a sample of children, adolescents, or adults who had experienced childhood interpersonal and/or non-interpersonal trauma. Of the 48 eligible studies, 29 referred to trauma of an interpersonal nature, 12 to trauma of a non-interpersonal nature, and 7 to overall trauma. Regarding the relationship between childhood trauma and AM, 24 studies found a negative relationship between childhood interpersonal trauma and AM; among the articles on non-interpersonal trauma, 10 studies found no relevant relationship; in the studies on overall trauma, 4 articles found negative relationship between overall trauma and AM. The literature explored in our systematic review supports the prevalence of a negative relationship between interpersonal childhood trauma and AM. This relationship is present regardless of psychiatric disorders (e.g., Depression, Post Traumatic Stress Disorder, and Personality Disorders), and in the presence of the latter, AM results even more fragmented. Future research should use more accurate methodologies in identifying and classifying childhood trauma in order to more precisely determine its effect on AM.

## Introduction

In the fifth version of the *Diagnostic and Statistical Manual of Mental Disorders* (5th ed.; DSM-5; American Psychiatric Association, 2013) criteria, childhood trauma is defined as exposure to actual or threatened death, serious injury, or sexual violence, occurring before age 18 (Teicher and Samson, 2013). This includes experiences of direct exposure to trauma, witnessing trauma, or learning about a trauma that happened to a friend or close relative. Some examples of childhood trauma may be motor vehicle accidents, bullying, terrorism, exposure to war, childhood maltreatment (physical, sexual, and emotional abuse, neglect), and exposure to domestic violence, that can overwhelm the individual’s psychic ability to respond to them adaptively (De Bellis and Zisk, 2014; Woodbury, 2019).

Childhood trauma is configured as interpersonal when includes physical/emotional/ sexual abuse, physical/emotional neglect and/or overprotection, including domestic violence, bullying, and violence in institutional settings, caused by perpetrators known or unknown; instead non-interpersonal trauma is configured as a, traumatization caused by non-human forces (e.g., accidents, natural disasters, diseases) (Musicaro et al., 2020; Baker et al., 2021; Thomas et al., 2021; Maharaj et al., 2022). In this systematic review we refer to overall trauma when both interpersonal and non-interpersonal trauma occurred in the same sample.

Worldwide, 10–30% of children and adolescents experiences at least an interpersonal trauma, such as sexual abuse, emotional abuse, or neglect (Stoltenborgh et al., 2015; Haselgruber et al., 2021), representing a risk factor for physical and chronic mental health conditions (Banker et al., 2019; McKay et al., 2021), especially if persistent over time (Read et al., 2005). It, indeed, can be linked to psychotic experiences (Croft et al., 2019), Depression (Yap et al., 2014), and Bipolar Disorder (BD) (Marangoni et al., 2016) in adulthood.

In addition, a meta-analysis by Masson et al. (2015) showed the association between childhood maltreatment with worse cognitive functioning in adulthood, both in healthy and clinical subjects in domains, such as working memory, executive function, attention, general intelligence, and impairment of social functioning. Furthermore, the results of both systematic literature reviews and meta-analysis by Fares-Otero et al. (2023a,b) highlighted the association between child maltreatment and impaired interpersonal relationships, specifically in individuals with Psychotic Disorders and Affective Disorders.

Childhood trauma is, in fact, associated with alterations in the structure, function, and connectivity of brain areas involved in cognition, such as the prefrontal cortex, hippocampus, and amygdala, and changes in white matter tract integrity, especially in the corpus callosum (Barczyk et al., 2023).

Traumatic experiences in childhood can have a profound impact on attachment behaviors (Cohen et al., 2017), depriving children of developing a healthy attachment with a primary caregiver (Stinehart et al., 2012; Meyer et al., 2013; Toof et al., 2020), with negative consequences as general emotional dysregulation (Zdankiewicz-Ścigała and Ścigała, 2020). This occurs due to stress-induced neurodevelopmental changes. There is evidence that exposure to severe stress during development can generate hormonal dysregulations and the functioning of the hypothalamic–pituitary–adrenal axis, which plays a central role in the body’s response to stress (Malaspina et al., 2008; Schore, 2009). This process can make individuals’ mental health vulnerable to stress and may put them at greater risk for future psychiatric illnesses and emotional disorders (McKay et al., 2021; Fares-Otero and Martinez-Aran, 2022). Moreover, both insecure attachment styles and early childhood trauma can lead to the development of alexithymia and dissociation (Liotti and Farina, 2016; Zdankiewicz-Ścigała and Ścigała, 2020), impacting more heavily on the development of affective, cognitive, behavioral deficits and impairment of social functioning, particularly the impairment of interpersonal relationships (Fares-Otero et al., 2023a,b), and social cognition (Rokita et al., 2018; Rodriguez et al., 2021), leading finally to the development of psychopathology in childhood and adulthood (Zdankiewicz-Ścigała and Ścigała, 2020), such as Psychotic and Affective Disorders (Fares-Otero et al., 2023a,b).

In addition to the consequences highlighted above, another negative effect of childhood trauma may be the unspecific recall of autobiographical memories (Williams, 2006; Williams et al., 2007; Hitchcock et al., 2014a; Ono et al., 2016; Barry et al., 2018), particularly evident in cases of individuals suffering from Major Depressive Disorder (MDD), BD and Post Traumatic Stress Disorder (PTSD) (Williams et al., 2007).

Autobiographical Memory (AM) can be defined as a form of explicit memory that involves events from one’s past that are personally relevant (Conway, 2009; Squire and Dede, 2015; Thome et al., 2020). AM incorporates both the episodic and semantic components of explicit memory. The episodic components of the AM involve memories of experiences determined in time and space and emotionally characterized; these types of memories imply a subjective sense of personal continuity over time (Tulving, 1987; Thome et al., 2020). Semantic components, instead, refer to general sense information about reality that does not imply spatiotemporal coordinates (Willoughby et al., 2012; Thome et al., 2020).

In the condition in which a reduced specificity in the recall of autobiographical memories occurs, literature refers to it as an Overgeneral Memory (OGM; Williams, 2006; Williams et al., 2007; Hitchcock et al., 2014a). This construct is based on Conway and Pleydell-Pearce’s (2000) theorization of AM. According to this model, autobiographical mnemonic traces would be organized into a series of structures, in which there would be a more general level where broad periods of an individual’s life are collected; at an intermediate level, memories of repeated events would be represented, and at the last level are the temporal and specific details of a well-known event (Conway, 2005; Hitchcock et al., 2014a). The processes of autobiographical memory retrieval are generative and direct. Contrary to this last one, generative retrieval is a top-down process that involves descending the memory hierarchy from these intermediate representations, which correspond to memories that are too general, to access event-specific knowledge (Sumner, 2012). The OGM would result from an interruption of this process (Williams et al., 2007; Hitchcock et al., 2014a) so that the event-specific memory cannot be reached, also using a clue.

The CaR-FA-X model is the most comprehensive model of the mechanisms underlying OGM (Williams, 1996; Williams, 2006; Williams et al., 2007) hindering the top-down process, also in childhood trauma. According to this model, OGM can occur when the generative recovery search process is prematurely terminated because of one or more of the following three mechanisms (Williams et al., 2007). Functional Avoidance (FA), in which the negative effect of information about a traumatic event is functionally avoided. More specifically, FA refers to the avoidance of the retrieval of specific memories as a means of affect regulation. It is suggested that this mechanism may emerge following exposure to early aversive experiences so that the person can avoid retrieving specific memories of aversive experiences. A less focused retrieval method is believed to be less detrimental to functioning because it reduces the effect of potentially upsetting information. But when this method is rigidly applied to every memory, it is believed to become maladaptive (Sumner et al., 2014). Capture and Rumination (CaR) occurs, instead, when a subject tries to recall positive and non-trauma-related events; he/she may activate traumatic memories semantically associated with them, interrupting the non-traumatic memory retrieval process. Thus, the CaR mechanism refers to when relevant conceptual information “captures” cognitive resources and interrupts the retrieval of a specific memory (Sumner et al., 2014). As a result, people also represent themselves more generally for positive aspects such as being liked or helpful to others (Sumner, 2012). Finally, it is also possible that such processes are implemented by Poor Executive Control (X), when subjects are unable to inhibit other semantically related memories and when they are unable to simultaneously keep in mind all the details related to memory (Hitchcock et al., 2014a; Barry et al., 2018).

In addition, according to Griffith et al. (2016), FA, CaR and X processes can be used to cope with the negative emotional consequences of trauma.

However, although the relationship between childhood trauma and AM has long been studied, there are, to date, conflicting results. Moore and Zoellner (2007), in their systematic review of the literature on the relationship between trauma exposure and re-enactment of autobiographical memories, verified that not all trauma-exposed subjects exhibit low specificity in the re-enactment of autobiographical memories. Furthermore, they found no difference between subjects who experienced trauma during childhood or adulthood. The authors verified that overgeneration is associated with an increased risk for the development of future depressive episodes and the development of PTSD following trauma. To better investigate memory, the authors recommend, among other things, designing studies in which it is analyzed in more detail whether the trauma was experienced during childhood or adulthood and the severity of the traumatic event.

Conversely, in the review conducted by Ono et al. (2016), it was highlighted that a reduced specificity of autobiographical memories is related to exposure to traumatic events in contrast to those who had not experienced traumatic events. In particular, a life history accompanied by trauma may negatively influence subjects’ ability to recall specific details of personal memories associated with negative emotions. According to the authors, their findings support Williams (1996) idea that OGM might be an acquired memory style for coping with childhood trauma. The authors suggest that individuals with a history of trauma manage negative emotions by remembering a low level of specific negative memories, thus avoiding them.

Barry and colleagues, in their 2018 systematic review, in contrast to the findings of Moore and Zoellner (2007), offer partial support for the findings of Ono et al. (2016). According to the authors, exposure to trauma is sufficient to impair the specificity of AM. However, the authors noted that participants with trauma exposure were more likely to recall fewer specific memories than controls if they had experienced the trauma during adulthood, rather than during childhood. The authors explain these results by considering that in minors, as more time elapsed between the time the trauma was experienced and the AM assessment, the effects of the trauma may have diminished over time. Another explanation could be that children are less competent in fully understanding the meaning and consequences of trauma than adults. As this result is at odds with Moore and Zoellner’s (2007) conclusion that trauma experienced in childhood might impair memory specificity, Barry et al. (2018) point out that this result should be further verified by analyzing not only when the trauma occurred, but also how it was processed and interpreted at that time. Therefore, analyzing the type of trauma experienced at a younger age would help us to understand whether this factor can partly explain the mixed results in the differences in the effects of trauma on biographical memory recorded in previous studies. Indeed, as Barry et al. (2018) note, there is a difference in how adults and children are susceptible to specific types of traumas. Therefore, it is important to investigate the possible effects of deficits in retrospective recall concerning specific types of traumas and their severity of trauma.

Considering these conflicting results, we conducted a systematic literature review to explore the relationship between childhood trauma and AM in children, adolescents, and adults, considering potential features in this relationship path, such as the type (interpersonal vs. non-interpersonal) and the presence of the mental disorder (clinical vs. healthy individuals).

## Materials and methods

### Search strategy

The current review was carried out following the Preferred Reporting Items for Systematic Reviews and Meta-analyses (PRISMA statement) guidelines (Moher et al., 2009; Page et al., 2021). The search strategy was conducted exclusively in the following three electronic databases: PubMed, Scopus, and Web of Science. We only considered studies published since 2014 investigating the relationship between childhood trauma and AM, in children, adolescents, and adults, since the latest similar review dates to 2014 (Hitchcock et al., 2014a). The search strategy was carried out from January 2023 to March 2023. The keyword combinations used for the search are as follows: “autobiographical memory” AND (“childhood trauma” OR “children trauma” OR “child trauma” OR “developmental trauma”), “autobiographical memory” AND (“childhood PTSD” OR “children PTSD” OR “child PTSD” OR “developmental PTSD”), “autobiographical memory” AND (“childhood post-traumatic stress disorder” OR “children post-traumatic stress disorder” OR “child post-traumatic stress disorder” OR “developmental post-traumatic stress disorder”). The study selection included two stages. In the first step, three reviewers (G.B., A.L.Z., C.S.) independently screened each record and each report retrieved for abstract and title. The articles were included or excluded according to their title and abstract; any disagreements were resolved through discussion with another independent reviewer (V.C.). Following this, in the second step, three reviewers (G.B., A.L.Z., C.S.) independently read and assessed the whole text of the eligible studies; any disagreements were resolved through discussion with another independent reviewer (V.C.). Any automation tools used in the process. Screening was done using Microsoft Excel.

### Inclusion and exclusion criteria

According to the PICO Framework, studies were included if they: (1) (P) were conducted in children, adolescents, and adults in a healthy and clinical sample; (2) (I) assessed the presence of childhood trauma, defined as exposure to actual or threatened death, serious injury or sexual violence, measured as interpersonal or non-interpersonal trauma (DSM-5; American Psychiatric Association, 2013), occurring before age 18 (Teicher and Samson, 2013); (3) (C) compared individuals without childhood trauma within the same sample of individuals (clinical or healthy sample), or compared to individuals in clinical o control group; (4) (O) assessed AM functioning with or without validated instruments; (5) qualitatively or quantitatively examined and reported associations between childhood trauma (exposure variable) and AM functioning (outcome variable).

We identified nine exclusion criteria for the articles screened for abstract and title, and they were excluded when: (a) they were review papers, (b) they were studies that commented on other studies, (c) they were studies that did not involve human beings, (d) they were editorial essay, (e) they were duplicates in the search of other databases, (f) they were studies that did not focus on the topic of interest (the relationship between AM and childhood trauma), (g) they were theoretical articles, (h) they were not written entirely in English, and (i) the full article was not available.

From eligible studies, articles were excluded when: (a) they did not measure all variables of interest (i.e., AM and childhood trauma), (b) they were studies assessing the efficacy of interventions, and (c) they had a different focus than the review’s purpose.

### Identified studies

**Figure 1:**
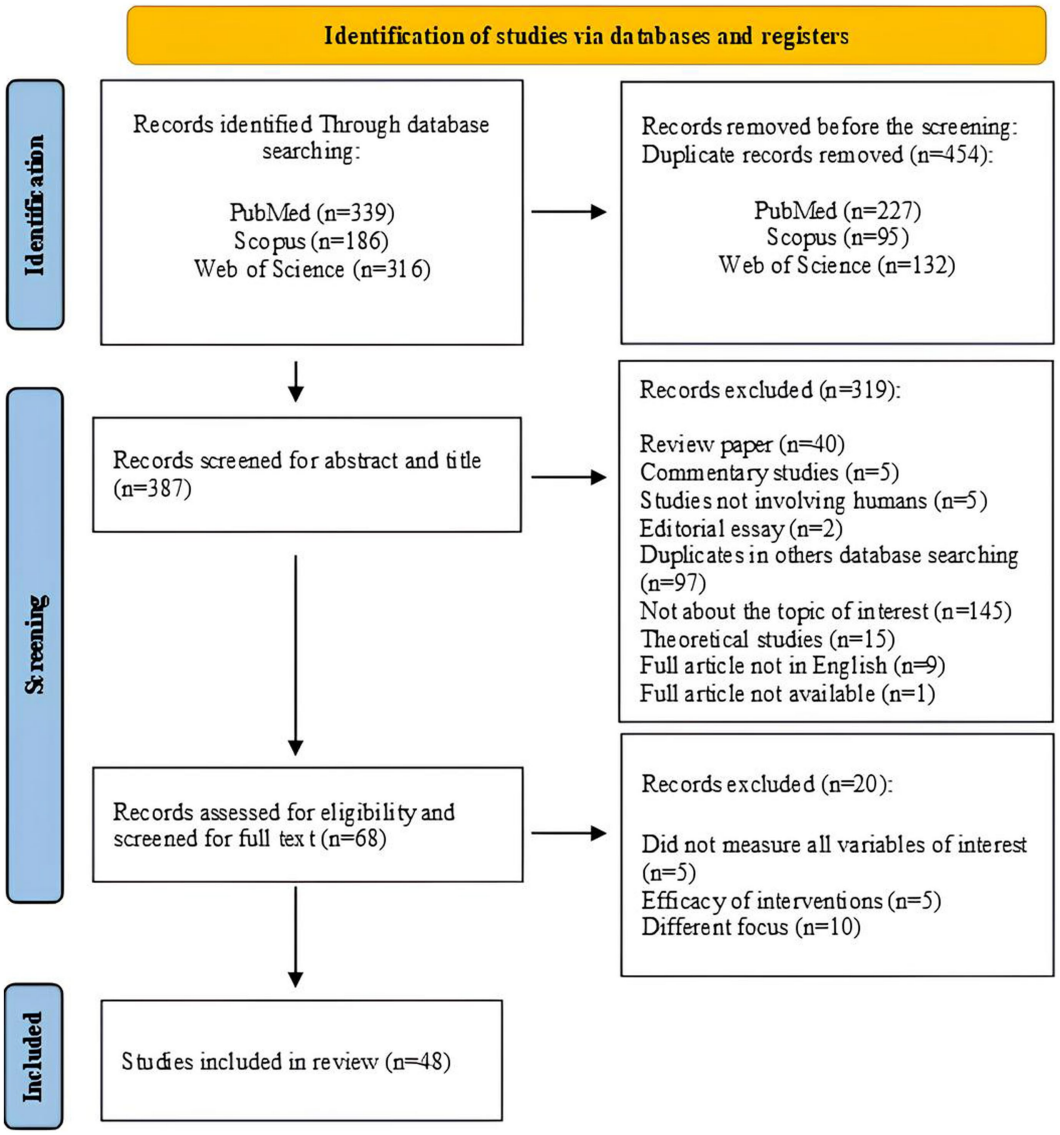
PRISMA flowchart of systematic review procedure.

Firstly, the search in the three databases identified a total of 841 studies: 339 were identified on PubMed, 186 on Scopus, and 316 on Web of Science. From these, 454 duplicates were detected in the three databases and were excluded. Thus, 387 articles resulted, and the reviewers screened their titles and abstracts and excluded 319 studies that met the exclusion criteria. Therefore, 128 resulting studies were assessed for eligibility. In this stage, the reviewers read the full articles and excluded 20 studies. Finally, 48 studies, that fell within the inclusion criteria, were assessed as eligible and included in the review. For more details, see Figure 1.

### Study outcomes

AM can be defined as “The ability to remember personal events is at the heart of what defines an individual as a person with obligations, roles, and commitments in a given society. It enables us to draw lessons from our past and plan our personal future. It helps us to orient and participate in complex social communities. Autobiographical memory is therefore crucial for a sense of identity, continuity, and direction in life.” (Berntsen and Rubin, 2012, p. 1).

After study selection, we categorized the study outcomes into positive relationship, no relationship and negative relationship between childhood trauma and AM.

We refer to positive relationship when there is an increase of AM quality, in terms of more definition, vividness, and details, to negative relationship when there is an impairment of AM, which is a decreased quality of memories in terms of less definition, vividness, and details, and to no relationship when there is no increase or decrease in AM quality.

### Data extraction

Data from eligible studies were extracted and tracked in Microsoft Word by three independent reviewers (G.B., A.L.Z., C.S.); discrepancies were resolved through consensus with an additional reviewer (V.C.) to ensure high quality of data extraction. For each article included in the review, the reviewers manually extracted information about the author and publication year, nationality, type of study (cross-sectional, longitudinal, qualitative, quantitative, quantitative on secondary data), sample size, diagnosis descriptive (N or % if reported), mean age (SD) or range in years, sex (N or %), instruments for childhood trauma and type (interpersonal, non-interpersonal, overall), instruments for outcome (AM), results about the relationship between a type of trauma and outcome (with statistical results) including covariates investigated in the included studies. Four independent reviewers (G.B., A.L.Z., C.S., V.C.) assessed the risk of bias and the quality of studies included in the systematic review conducted using the Newcastle-Ottawa Scale (NOS) (Wells et al., 2014). The data extracted from the articles were reported in the summary table of the studies included in the review (Supplementary Table S1).

## Results

### Study inclusion and characteristics

From 48 selected studies, 29 were characterized by participants only with interpersonal childhood trauma, 7 analyzed samples with overall types of childhood trauma, and 12 were characterized by non-interpersonal childhood traumatic experiences, such as war trauma or natural catastrophes. The total sample of studies included in the review consisted of 57,887 individuals (sample size range 26–23,807). Among all the studies analyzed in this review, 35 articles included healthy samples, 2 clinical samples, and 11 both clinical and healthy samples. 29 studies retrospectively assessed childhood trauma in adult samples, 14 assessed it in samples of children under 18 years old, and 5 included both adult and child samples. Almost all studies, specifically 32, used validated instruments to measure childhood trauma, 9 used *ad hoc* instruments, and 2 mixed instruments (validated and *ad hoc*). In 5 studies trauma was not assessed, because it was already known. The most common instrument used to assess childhood trauma, concerning traumatic life experiences in childhood, was the Childhood Trauma Questionnaire (CTQ; Bernstein and Fink, 1998). Even regarding the assessment of AM, most of the studies, particularly 32, used validated instruments, 15 *ad hoc* instruments, and 1 mixed instrument (validated and *ad hoc*). The most widely used tool for AM was the Autobiographical Memory Test (AMT; Williams and Broadbent, 1986), which assesses autobiographical memories in response to cue words.

### Study quality assessment

The Newcastle-Ottawa Scale (NOS; Wells et al., 2014), a quality assessment tool that rates the risk of bias in non-randomized studies, has been used for all included studies (see supplementary materials). The included studies’ mean quality rating (range between 0 and 9) was 6.31 (*SD* = 1. 64), a range of 3–9. Overall, 6 (12.5%) studies were rated as ‘poor’ (NOS score = 3–4), 10 (20.8%) studies were rated as ‘fair’ (NOS score = 5), 11 (22.9%) studies were rated as ‘good’ (NOS score = 6), and 21 (43.8.%) studies received a rating considered as ‘high’ (NOS score > 6) (Huntjens et al., 2014; Neshat Doost et al., 2014; Harris et al., 2016; Parlar et al., 2016; Wang et al., 2016; Wittekind et al., 2016; Peltonen et al., 2017; Saleh et al., 2017; Wittekind et al., 2017; Feurer et al., 2018; Kaczmarczyk et al., 2018; Tian et al., 2018; Viard et al., 2019; Jiang et al., 2020; Barry et al., 2021; Bendstrup et al., 2021; Chiasson et al., 2022; D’Amico et al., 2022; Pacheco and Scheeringa, 2022; Thomson and Jaque, 2022; Fishere and Habermas, 2023). The representativeness of samples was mixed (clinical and healthy samples), including children, adolescents, and adults, and most of the included studies did not report either on non-response or *a priori* power analyses or otherwise justified their sample sizes. More than half of the included studies (*N* = 29) controlled for covariates in their design or analysis (i.e., age, gender) and 19 studies used a control group for childhood trauma (Huntjens et al., 2014; Neshat Doost et al., 2014; Berthelot et al., 2015; Harris et al., 2016; Parlar et al., 2016; Wang et al., 2016; Wittekind et al., 2016; McCrory et al., 2017; Saleh et al., 2017; Wittekind et al., 2017; Kaczmarczyk et al., 2018; Tian et al., 2018; Viard et al., 2019; Jiang et al., 2020; Barry et al., 2021; Bendstrup et al., 2021; Chiasson et al., 2022; Pacheco and Scheeringa, 2022; Fishere and Habermas, 2023) (see Supplementary Table S1).

### Association between childhood trauma and AM

Studies investigating the effects of childhood traumatic events on children, adolescents, and adults’ cognitive functioning have found contrasting results regarding the effects of earliest memories (AM) and specific autobiographical memories (Williams, 1996, 2006; Williams et al., 2007). We aimed to investigate the relationship between childhood trauma and AM by regarding this relationship considering two features: the type of trauma (interpersonal and non-interpersonal) and the presence of any mental disorders (e.g., Depression, PTSD, and Personality Disorders).

In the exploration of this relationship, some research focused on war trauma (Wittekind et al., 2016; Fohn et al., 2017; Peltonen et al., 2017; Wittekind et al., 2017), on natural catastrophes (Weems et al., 2014; Dawson and Bryant, 2016; Tian et al., 2018), on terroristic attacks (Vallet et al., 2017), on accidents (Hitchcock et al., 2014b; McKinnon et al., 2017), and on stressful medical care (McKinnon et al., 2017; Risløv Staugaard et al., 2017; Goldfarb et al., 2019), while others on interpersonal trauma such as neglect, sexual abuse, maltreatment, parental separation, parental bereaved or overprotection (Crane et al., 2014; Huntjens et al., 2014; Neshat Doost et al., 2014; Berthelot et al., 2015; Kaynar and Er, 2015; Griffith et al., 2016; Harris et al., 2016; Varnaseri et al., 2016; Wang et al., 2016; McCrory et al., 2017; Kaczmarczyk et al., 2018; Hawkins et al., 2020; Jiang et al., 2020; Barry et al., 2021; Bendstrup et al., 2021; Ding and He, 2021; Hakamata et al., 2021; Lawson et al., 2021; Salomão et al., 2021; Alaftar and Uzer, 2022; Chiasson et al., 2022; D’Amico et al., 2022; Lin et al., 2022; Thomson and Jaque, 2022; Wolf and Nochajski, 2022; Zhu and Hakim-Larson, 2022; Fishere and Habermas, 2023; Goldfarb et al., 2023; Zhang et al., 2023). Other studies assessed both non-interpersonal and interpersonal trauma within the same sample (Parlar et al., 2016; Saleh et al., 2017; Feurer et al., 2018; Staniloiu et al., 2018; Viard et al., 2019; Pacheco and Scheeringa, 2022; Kangaslampi, 2023).

### Non-interpersonal childhood trauma

Most studies that have examined non-interpersonal childhood trauma agree about the absence of negative relationship between childhood trauma and AM, specifically there was no decreased ability to recall and describe specific events or earliest memories (Weems et al., 2014; Hitchcock et al., 2014b; Dawson and Bryant, 2016; Wittekind et al., 2016; Fohn et al., 2017; McKinnon et al., 2017; Peltonen et al., 2017; Risløv Staugaard et al., 2017; Wittekind et al., 2017; Goldfarb et al., 2019). The absence of this negative relationship was found in healthy and clinical samples.

### Non-interpersonal childhood trauma in healthy samples

Studies with samples of healthy individuals found that children and adolescents exposed to trauma (e.g., war trauma, hurricanes) did not show OGM (Peltonen et al., 2017), indeed, even more vivid memories and accuracy of stressful childhood events (Weems et al., 2014; Dawson and Bryant, 2016; Risløv Staugaard et al., 2017), also in the presence of current psychological and physical symptoms (i.e., PTSD, Depression) (Hitchcock et al., 2014b; McKinnon et al., 2017; Goldfarb et al., 2019), as well as fear of past traumatic events in adults (Fohn et al., 2017). Furthermore, Weems et al. (2014) revealed that, although there was no relationship between childhood trauma (Hurricane Katrina) and poorer specific memories trauma-related, a high exposition to another similar traumatic event (Hurricane Gustav) leads to a reconsolidation of the memories, while a low post-reactivation of a similar traumatic event slowed down the initial memory reconsolidation, that is few memories of the first traumatic event. Fewer studies, instead, found a decreased quality of memories in terms of definition, vividness, and details in adolescents (Tian et al., 2018) and youngest compared to older adults (Vallet et al., 2017).

### Non-interpersonal childhood trauma in clinical samples

No relationship between childhood trauma and AM has also been found in studies that have examined non-interpersonal trauma in adult clinical samples. Wittekind et al. (2017) did not find differences in the ability to remember past experiences in traumatized PTSD adults, compared both to the control group (without trauma and PTSD) and traumatized without PTSD.

### Overall trauma

Studies that considered both interpersonal and non-interpersonal trauma found mixed results regarding the relationship between childhood trauma and AM, both in healthy and clinical samples.

### Overall trauma in healthy samples

The studies that considered healthy samples with overall trauma focused specifically on children. The study of Kangaslampi (2023), for example, noted no differences in the oldest and most recent memories, relative to the specificity of AM, in trauma and accident-related content, or emotional content in children. However, the study by Pacheco and Scheeringa (2022) highlighted that children who had experienced repeated traumatic events or Hurricane Katrina reported their trauma memories in less detail, compared to children who had experienced a single episode of trauma. In addition, Feurer et al. (2018) found an effect on the cue’s valence, evidencing that the mother’s high levels of stressful life events predicted a decrease in children’s AM for positive cues, but not for negative cues.

### Overall trauma in clinical samples

Among the studies that have considered overall traumas in clinical samples, only one study considered children, showing that PTSD patients had significantly lower performance for both immediate and delayed recall than controls (Viard et al., 2019). Looking for adult samples, two studies revealed any association between childhood trauma experiences and AM regardless of the presence of depression (Parlar et al., 2016; Saleh et al., 2017). However, Staniloiu et al. (2018) analyzed 28 adult patients with the diagnosis of Dissociative Amnesia (retro-anterograde), revealing that 25 out of 28 patients suffered different types of traumas in childhood (car accidents, previous history of sexual abuse, professional failures, stressful events). In this study, depressive symptoms and mild traumatic brain injuries were also common findings, suggesting “a mechanism of incubation of trauma or kindling desensitization” (p. 144) in subsequent AM.

### Interpersonal childhood trauma

Interpersonal traumatic experiences (i.e., neglect, maltreatment, sexual abuse, parental bereavement) could have a higher impact on recalling memories than other types of traumatic experiences (i.e., war trauma or accidents). Trying to systematize results about the relationship between interpersonal traumatic experiences and recalling of autobiographical memories, we found contrasting results.

Specifically, some studies did not find any difference in AM and subtype of episodic memory in the presence of childhood traumatic experiences (D’Amico et al., 2022; Thomson and Jaque, 2022; Zhang et al., 2023); others, instead, sustained the prominent relationship between interpersonal childhood traumatic experiences and a cognitive impairment, with a decreased ability to recall specific episodes and earliest autobiographical memories, in terms of details accuracy, lack of emotion and cognitive terms, and OGM (Crane et al., 2014; Neshat Doost et al., 2014; Berthelot et al., 2015; Harris et al., 2016; Varnaseri et al., 2016; McCrory et al., 2017; Hawkins et al., 2020; Ding and He, 2021; Hakamata et al., 2021; Salomão et al., 2021; Alaftar and Uzer, 2022; Lin et al., 2022; Wolf and Nochajski, 2022; Fishere and Habermas, 2023; Goldfarb et al., 2023), regardless of the PTSD, of Dissociative Identity Disorder (DID), of Mild Cognitive Impairment (MCI), of MDD, of Borderline Personality Disorder (BPD) or Schizophrenia diagnosis (Huntjens et al., 2014; Griffith et al., 2016; Wang et al., 2016; Kaczmarczyk et al., 2018; Jiang et al., 2020; Barry et al., 2021; Bendstrup et al., 2021; Chiasson et al., 2022). Finally, others sought to evaluate the impact of specific disorders in such retrieval (Huntjens et al., 2014; Griffith et al., 2016; Wang et al., 2016; Kaczmarczyk et al., 2018; Jiang et al., 2020; Barry et al., 2021; Bendstrup et al., 2021; Chiasson et al., 2022).

### Interpersonal childhood trauma in healthy samples

Related to interpersonal trauma in studies with healthy children’s populations, Berthelot et al. (2015) found that children exposed to abuse/neglect displayed poorer cognitive performance in episodic memory and executive functions of initiation, compared to children and adolescents non-exposed. Specifically, concerning AM, several studies showed a strong relationship between childhood traumatic experiences and OGM (Crane et al., 2014; Neshat Doost et al., 2014; McCrory et al., 2017; Salomão et al., 2021). For example, Crane et al. (2014) showed that in a sample of 5,792 early adolescents, aged 13 years, childhood trauma was associated with the presence of OGM. However, when the trauma was moderate, the OGM decreased slightly compared to severe trauma. In addition, a recent study revealed that older children provided more unique details than younger children, and females also provided more unique details than males (Lawson et al., 2021).

Regarding the adult populations, similarly to adolescents and children’s samples, Lin et al. (2022), in a sample of 6,466 Chinese adults gender-balanced, found a global cognitive decline over time (including episodic memory and executive functions) after two or more deprivation-related childhood traumatic events, such as emotional neglect, household mental illness, incarcerated household member, parental separation or divorce, and parental death. In the same vein, Hakamata et al. (2021) found that when childhood trauma was more severe, the OGM was greater in adults. Specifically, semantic-associated memory, a type of OGM, was greater: participants remembered more semantic details and not contextual details of the traumatic event. In addition, Zhang et al. (2023) compared Chinese individuals with or without adverse childhood experiences in the manifestation of cognitive functions, specifically episodic memory, revealing that individuals who experienced four or more adverse childhood experiences were more likely to have decreased global cognition, but not specifically in episodic memory. Supporting this study, Kaynar and Er (2015) found that severe childhood trauma was associated with more detailed memories of negative events and OGMs of recent and positive events. Again, Zhu and Hakim-Larson (2022), in a Canadian sample of 204 participants, observed that narratives of maltreatment experiences were more consistent and contained more information than those of positive events. In contrast, Fishere and Habermas (2023) evidenced that narratives of maltreatment in emerging adults, who have experienced repeated maltreatment in childhood (physical and sexual abuse or neglect), contained fewer emotional statements compared to the control group. Interesting results were obtained by Goldfarb et al. (2023), who interviewed 115 adults 2 decades after a documented abuse when they were 3–16 years old. Although the authors did not examine adults’ memory of real abuse, they assessed the accuracy of reports relevant to historic child abuse through a standard forensic interview, the cognitive interview with mental reinstatement, or the cognitive interview with mental and physical-context reinstatement. Findings revealed that what the adults remembered about the abuse was accurate, varying mostly in quantity. The authors underlined the importance of the interview type used, which could explain such differences in quantity.

### Interpersonal childhood trauma in clinical samples

Beyond the evidence that raised the negative relationship between interpersonal traumatic experiences and AM, studies underlined it in adults regardless of the Personality Disorder, PTSD, or Depression diagnoses.

In a study of patients with MDD, Griffith et al. (2016) found that only childhood physical abuse was related to fewer specific autobiographical memories, while childhood sexual abuse was not significantly related to AM, after controlling PTSD and MDD. In contrast to this study, Wolf and Nochajski (2022), in a sample of 297 participants, aged between 18 and 73 years, highlighted that childhood sexual abuse was associated with a reduction in AM. Furthermore, Chiasson et al. (2022) revealed the presence of the relationship mentioned above regardless of PTSD. In this regard, a study by Harris et al. (2016) examined individuals with or without experiences of childhood sexual abuse on the ability to recall specific memories, hypothesizing that, rather than the trauma itself, avoidance strategies were the cause of the poor recovery of autobiographical information. Results confirmed their hypothesis in that avoiding was associated with less specific autobiographical memories regardless of childhood trauma condition. According to such results, in participants who experienced interpersonal childhood traumatic experiences, the presence of Depression or PTSD was not associated with the difficulty of recalling specific memories, but the presence of such mental disorders hinders recovery due to the avoidance strategies used.

In a recent study, Kaczmarczyk et al. (2018) examined not only childhood sexual abuse, but also other forms of childhood maltreatment, such as emotional abuse, physical abuse, physical neglect, and emotional neglect, in a clinical sample with MDD and control sample. The authors showed that it was the severity of childhood trauma that explained the reduction of AM specificity and not the severity of depressive symptoms. Contrarily, Saleh et al. (2017), comparing two groups of 64 antidepressant-free depressed and 65 never-depressed individuals on cognitive functions (i.e., working and episodic memory and processing speed) and childhood overall traumatic experiences, revealed that, although the depressed group exhibited poorer performance in episodic memory, those performances were not predicted by childhood traumatic experiences. However, it should be considered that, unlike the study by Kaczmarczyk et al. (2018) which focused on interpersonal trauma, Saleh et al. (2017) analyzed a wide range of interpersonal and non-interpersonal trauma.

Generally, as regards clinical samples, studies that assessed the relationship between childhood trauma and AM showed similar results to non-clinical samples (Huntjens et al., 2014; Griffith et al., 2016; Wang et al., 2016; Kaczmarczyk et al., 2018; Jiang et al., 2020; Barry et al., 2021; Bendstrup et al., 2021; Chiasson et al., 2022).

For example, a recent study conducted by Bendstrup et al. (2021), on female participants suffering from BPD, showed, beyond their inconsistency in autobiographical memories compared to the control group, already documented in the literature for individuals with BPD diagnosis (Jørgensen et al., 2012; Rasmussen et al., 2017), the association between childhood traumatic experiences (including emotional, physical and sexual abuse, emotional and physical neglect) and reduced narrative coherence.

Barry et al. (2021), however, showed that a greater number and more severe adverse childhood experiences were associated with reduced AM in participants, but that those with a diagnosis of Schizophrenia retrieved fewer specific memories than control participants (without a diagnosis) regardless of the presence of severe adversity experienced in childhood.

## Discussion

In this review, we systematize studies that explore the relationship between childhood trauma and AM in children, adolescents, and adults, considering the period from 2014 to 2023.

To our knowledge, no review study in the literature dealt with the specific relationship between AM and childhood trauma considering potential features in this relationship path, such as the type (interpersonal vs. non-interpersonal) and the presence of the mental disorder (clinical vs. healthy individuals).

As we argued in the introduction, previous studies in this field gave mixed results regarding the relationship between childhood trauma and AM. Our article aimed to explore this relationship considering the type of trauma and the presence of mental disorder. We divided the selected studies according to the type of childhood trauma experienced, classifying them into interpersonal trauma (i.e., neglect, sexual abuse, maltreatment, parental separation, parental bereaved or overprotection), non-interpersonal trauma (i.e., war trauma, natural catastrophes, terroristic attacks, stressful medical care), and overall trauma (interpersonal and non-interpersonal).

The results showed that of the 48 studies identified, 29 assessed interpersonal childhood trauma, 12 studies assessed non-interpersonal trauma, and 7 assessed overall trauma.

Regarding the assessment of the quality and risk of bias of the included studies conducted with NOS, it is useful to report that only 6 studies (12.5%) were characterized by low scientific rigor; in fact, they received a “poor” score indicative of low scientific quality. Among the included studies, 10 (20.8%) received a “fair” score meaning that they were characterized by discrete scientific rigor. In contrast, 11 studies (22.9%) received a “good” score and 21 studies (43.8%) received a “high” score, indicative of good and high scientific quality, respectively. Therefore, it can be seen that more than half of the included studies are characterized by adequate/high scientific rigor; however, the interpretation of these results should be cautious.

### Association between childhood trauma and AM

Most studies that have examined non-interpersonal childhood trauma agree about the absence of negative relationship between childhood trauma and AM, specifically there was no decreased ability to recall and describe specific events or earliest memories, such result was found in both healthy and clinical samples.

Instead, studies that considered both interpersonal and non-interpersonal trauma found mixed results regarding the relationship between childhood trauma and AM, both in healthy and clinical samples. Most of them found a negative relationship, that is an impairment of the AM, in terms of details and cue valence.

In the same vein, studies that considered interpersonal trauma showed contrasting results, anyway, most studies agree about the presence of a negative relationship between childhood trauma and AM, regardless of the presence of mental disorder.

### Non-interpersonal trauma in healthy and clinical samples

Of the 12 studies that assessed non-interpersonal childhood trauma, 10 agreed that there was no negative relationship between childhood trauma and AM. Instead, in the study by Tian et al. (2018), a negative relationship between earthquake trauma and OGM tendency was found. However, the instrument used by the authors to assess trauma was created *ad hoc* for the study and only included six items related to earthquake trauma. In this way, it was impossible to check whether other possible past traumas were present in the subjects’ history.

In this regard, as highlighted by Weems et al. (2014), if a trauma of the same nature was repeated, this influenced the memory of the first one. This result suggested the presence of possible additive effects of trauma to be considered when conducting trauma studies. It would be useful, in future studies, to highlight more clearly the possible traumatic history of the subjects to understand the possible presence of other types of traumas and their persistence over time.

### Overall trauma in healthy and clinical samples

Of the 7 studies considering overall trauma, in 4 a negative relationship between childhood trauma and AM was identified and in 3 (Parlar et al., 2016; Saleh et al., 2017; Kangaslampi, 2023) no relationship was found. In Parlar et al. (2016), for example, most of the participants were taking medication at the time of the AM assessment and this could be a potential intervening variable affecting the re-evocation of autobiographical memories. In the study by Saleh et al. (2017), a wide range of trauma types was assessed, without differentiating the effects of each and not considering their severity and chronicity. Therefore, these aspects should also be considered.

### Interpersonal trauma in healthy and clinical samples

Proceeding in the screening of the studies, of the 29 studies that dealt with interpersonal childhood trauma, 24 found a negative relationship between trauma and AM, while the other studies found no such relationship. Among these, two (D’Amico et al., 2022; Thomson and Jaque, 2022) found no association, and two (Kaynar and Er, 2015; Zhu and Hakim-Larson, 2022) found an association between trauma and increased trauma-related autobiographical memories. However, when analyzed more in detail, the results of these studies could be influenced by methodological aspects. For example, Zhu and Hakim-Larson (2022) found that there were coherent narratives of the maltreatment experienced in the sample. However, of the 204 participants, 120 (59%) reported current or previous psychotherapeutic and/or psychopharmacological treatment and showed greater narrative coherence than those who did not undergo treatment. Therefore, the detected effect on AM could be due to intervening variables that were not effectively controlled.

In most studies (24), instead, it was possible to detect the prominent presence of AM impairment, in terms of accuracy and quantity of details, lack of emotion and cognitive terms, and OGM, in healthy and clinical samples.

Most findings concerning interpersonal childhood trauma could be explained by the fact that the traumatic experience is embedded in the context of familial and/or extra-familial relationships. As mentioned above, individuals are designed to structure relationships of a social nature. There is evidence that the reactions and development of individuals who experienced childhood interpersonal trauma in the contexts of attachment and relationships follow different trajectories from those of individuals who experienced single-episode trauma in childhood or adulthood.

As well as childhood interpersonal trauma affects the AM and consequently the subjective sense of personal continuity over time, previous research (Fares-Otero et al., 2023a,b) recommended investigating the impact of childhood interpersonal trauma on social cognition, defined as “the mental operations that underlie social interactions, including perceiving, interpreting, and generating responses to the intentions, dispositions, and behaviors of others” (Green et al., 2008, p. 1211).

According to Fonagy et al. (2023), one of the key principles of attachment research is that the attachment strategy adopted in early life, in response to the quality of care received, influences the individual’s relational functioning in adulthood, creating an internal working model. It is important to consider the relationship between childhood attachment and subsequent social–emotional functioning, in terms of the significance of internal working models on social functioning in life. In this way, attachment can be considered as a mediator in the relationship between early trauma and the risk of psychopathology and functioning in future life.

Moreover, experiencing trauma of an interpersonal nature often means experiencing multiple traumas of different types. It is already demonstrated that the pathological and non-pathological developmental sequelae resulting from complex trauma (Kira et al., 2013) can differ significantly from those related to single-episode trauma.

Therefore, the literature explored in our systematic review supports the prevalence of a negative relationship between interpersonal childhood trauma and AM, independently from the presence of mental disorders, with all the necessary precautions with respect to the interpretation of the results. Nonetheless, the heterogeneity of results identified in the literature, as well as for our results, regarding the relationship between interpersonal childhood trauma and AM may be due - at least in part - to inaccurate detection of interpersonal childhood trauma. In fact, the qualitative analysis of the studies included in our review revealed the absence of important information in the detection of trauma such as, for example, persistence over time.

In this regard, Kira and colleagues (Kira et al., 2013; Kira, 2022) made a classification of trauma types, concretized in the Development-Based Trauma Framework (DBTF) model. According to this classification, childhood trauma can be defined as: type I, when it occurs in a single episode (i.e., car accident); type II, complex, caused by repeated similar episodes over time that no longer occur (i.e., child maltreatment); type III, complex, caused by repeated similar episodes over time that is still occurring (i.e., racial discrimination); type IV, cumulative, consisting of at least three traumas, that is a central trauma (sensitizing and conditioning responses to possible stressogenic events), a triggering trauma that triggers the post-cumulative response (“The last straw that breaks the camel’s back,” p. 182), and a peripheral trauma (trauma that is less salient in the totality of traumatic events experienced but can become salient when reactivated by similar traumatic events) (Kira et al., 2013). Recently, Kira (2022) also highlighted how the type, duration, frequency, and exposure modality (direct–indirect) have different effects on a person’s development. In this review, only two studies assessed the persistence of childhood trauma over time and found a significant difference in the impairment of AM (Lin et al., 2022; Pacheco and Scheeringa, 2022). In the studies reviewed, it was not possible to classify trauma according to the DBTF model. This classification would have allowed a better understanding of the relationships between trauma and impairment of AM.

### Strengths and limitations

This study has some limitations. The first limitation in this systematic review is that we only considered electronic databases in the search strategies. The second limitation of this research was that it was not preregistered in any database before starting the systematic review process. Third, forward and backward citation analysis was not conducted in this systematic review, i.e., no manual searches of articles cited by a publication or searches of articles citing a publication were conducted. In addition, no automation tools were used in the process. The interpretation of the results of the studies included in our systematic review was conducted qualitatively; therefore, the possible risk of bias cannot be excluded. Assessment of the quality and risk of bias of the studies included in our systematic review was conducted through the use of the NOS. However, this tool is based on a qualitative assessment that may be affected by the greater or lesser experience of those conducting the assessment. On the other hand, PRISMA requires an assessment of the risk of bias of the included studies; the use of the NOS allowed us to comply with PRISMA guidance and carry out an assessment of the quality and risk of bias of the included studies. Other strengths of this study include the rigorous methodology with the systematic search, study selection, and data extraction, which were all performed by independent researchers, the inclusion of all studies published in the period 2014–2023, the evaluation of the quality of each study, and other key practices for systematic reviews. Qualitative analysis of the studies included in this review can be very helpful in better understanding the relationship between the type of trauma and AM. The results included in this study may be useful in better understanding trauma in designing future research and planning more appropriate interventions for individuals who experience trauma.

### Practical and clinical implications

Research analyzed the relationship between interpersonal and non-interpersonal childhood trauma and AM, produced results that lead to the hypothesis that several trauma-related variables contribute to producing different effects on people who experience childhood trauma. Our systematic review highlights the scientific and clinical requirement to consider, in the assessment process, not only the occurrence of the traumatic experience but, according to Kira et al. (2013, 2014, 2021), assess the person’s trauma profile and global traumatic dynamics in his/her life, that is the subjective experiential pattern of exposure to single or multiple extreme stress adversities. Indeed, according to scientific literature on the topic, descriptive variables of the trauma construct, such as the type (interpersonal/non-interpersonal), age of exposure, perpetrator (known or unknown person, family member or non-family member), mode of exposure (direct/indirect), duration, frequency, would concur in determining and, thus, explain the heterogeneity of post traumatic clinical outcomes (Kira et al., 2013; Jowett et al., 2020; Kira, 2022; Sölva et al., 2023). So, this means that different profiles of trauma lead to different outcomes and comorbidity (Kira et al., 2014). Therefore, it is crucial to analyze post-traumatic clinical outcomes considering the interaction between these descriptive variables, as the effectiveness of treatment necessarily also depends on the accuracy of diagnostic framing. This increases the likelihood of helping the person to develop Post-Traumatic Growth (PTG), that is, an improvement in quality of life produced by the emotional and cognitive processing of trauma-related experiences (Vloet et al., 2017; Thomas et al., 2021).

In addition, the identification and acknowledgment of descriptive variables of trauma is also crucial for scientific research as this helps to improve the research design, including the choice of sample type, sampling technique, instruments to be used, and the type of statistical/descriptive analysis to be applied.

### Conclusions and future studies

Based on all the results observed in this review, it emerges strongly that interpersonal childhood trauma was associated with an impairment of AM, fundamental for a subjective sense of personal continuity over time (Tulving, 1987; Thome et al., 2020). This relationship is present regardless of psychiatric symptomatology and in the presence of the latter, AM results even more fragmented. Relating to the literature on the topic, we underline that, according to Williams (1996, 2006) and Williams et al. (2007), trauma experienced in childhood can impair AM to avoid negative memories related to the trauma and positive memories semantically associated with the trauma. We think that future research should use more accurate methodologies in the detection and classification of childhood traumas to determine the real effect of these on AM. Therefore, as also suggested by previous authors (i.e., Barry et al., 2018), it is essential to carry out studies that can accurately assess the type and severity of trauma. Using the above-mentioned DBTF model, it would be possible to identify type and severity, as well as overtime persistence (Kira et al., 2013; Kira, 2022). We consider that it is also necessary to examine whether the person, who experienced the childhood trauma, is currently or previously undergoing treatment (psychotherapeutic and/or pharmacological) and, in the context of interpersonal trauma, who is the perpetrator (intra-familial and/or extra-familial). A supplementary recommendation for future research could be the structuring of an instrument able to discriminate between the types of traumas.

## Data availability statement

The original contributions presented in the study are included in the article/Supplementary material, further inquiries can be directed to the corresponding author/s.

## Author contributions

GB: Conceptualization, Methodology, Writing – original draft, Writing – review & editing. ALZ: Conceptualization, Methodology, Writing – original draft, Writing – review & editing. CS: Conceptualization, Methodology, Writing – original draft, Writing – review & editing. VC: Writing – review & editing. RP: Project administration, Writing – review & editing, Supervision.
