# Supplementary material for: The relationship between childhood interpersonal and non-interpersonal trauma and autobiographical memory: a systematic review
Source: Front Psychol. 2024 Jan 17;15:1328835. doi: 10.3389/fpsyg.2024.1328835 (PMC10827865; doi:10.3389/fpsyg.2024.1328835)
Supplement: Supplementary file 1 [file Table_1.DOCX]

Supplementary Material

Table 1

*Summaries of studies*

| **Author/s** | **Nationality Sample** | **Type of Study** | **Type of Sample** | **Sample Size,**  **Age, Sex** | **Instrument to Assess Childhood Trauma** | **Type of Childhood Trauma** | **Instrument to Assess Autobiographical** | **Results** | **NOS**  **Score** |
| --- | --- | --- | --- | --- | --- | --- | --- | --- | --- |
| Alaftar & Uzer, 2022 | Turkey | Cross-sectional quantitative study | Healthy | N= 240  Mothers group (N=120): *M*_age_=49.93±4.56  120 F  Adolescents group (N=120): *M*_age_=21.78±1.50 96 F, 19 M | Turkish version of the Childhood Trauma Questionnaire (CTQ; Sar et al., 2012). | Interpersonal:  physical abuse, emotional abuse, sexual abuse, emotional neglect, and physical neglect. | Autobiographical Memory Recall Task ad hoc. | Overgeneral memory tendency regarding adverse childhood experiences intensified the relationship between adverse childhood experiences and children’s disconnection-rejection schemas transmitted by their mothers (*β*= 0.02, *SE*= 0.01, *t*= 2.11, *p*= .04; 95% *CI*^h:^ [0.01, 0.03]). | 5 |
| Barry et al., 2021 | Not specified | Cross-sectional quantitative study | Both Healthy and Clinical | N=120  Schizophrenia group (N=79): *M*_age_=44.62±12.74  Control group (N=41):  *M*_age_=37.80±12.38 | Maltreatment and Abuse Chronology of Exposure (MACE; Teicher & Parigger, 2015). | Interpersonal:  parental physical abuse, parental verbal abuse, parental non-verbal emotional abuse, familial and non-familial sexual abuse, witnessed physical violence towards parents, witnessing violence towards siblings, peer emotional violence, peer physical violence, emotional neglect and physical neglect. | Autobiographical Memory Test (AMT; Williams & Broadbent, 1986). | Participants who experienced a greater number of, and more severe, adversity had greater difficulty retrieving memories of specific autobiographical events (*b*= -0.21, *SE*= 0.10, 95% CI: [-0.41, -0.02], but both participants with schizophrenia with (*Ψ*= 6.292, 95% CI: [4.97, 7.49], *p <* .001) or without severe adversity (*Ψ*= 6.206, 95% CI: [5.12, 7.12], *p <* .001) retrieved fewer specific memories than controls, regardless of the presence of severe childhood adversity, after controlling for age, gender, education, depression symptoms, and verbal fluency. | 9 |
| Bendstrup et al., 2021 | Denmark | Cross-sectional quantitative study | Both Healthy and Clinical | N=54  BPD^b^ group (N=26): *M*_age_=28.67±7.21  Control group (N=28):  *M*_age_=28.88±8.77  54 F | The Danish version of the Childhood Trauma Questionnaire (CTQ; Kongerslev et al., 2019). | Interpersonal: emotional abuse, physical abuse, sexual abuse, emotional neglect, and physical neglect. | AM^g^ interviews ad hoc. | Participants with BPD showed incoherent autobiographical narratives (F(3, 141)= 4.060; p= .008) and more self-reported childhood adversity (t (48)= −7.502; p < .0001) compared to the control group, after controlling for age, subject education, parent education, Wechsler Adult Intelligence Scale Verbal Comprehension Index (WAIS VCI) score, and number of words in the autobiographical stories. | 7 |
| Berthelot et al., 2015 | Canada: Easter Quebec | Cross-sectional quantitative study | Healthy | N=236  High-risk offspring of a parent with schizophrenia or BPD group (N=66): *M*_age_=17.2±4.10  Control group (N=170): *M*_age_=16.1±4.51  120 F, 116 M | Childhood Adverse Life Events Chart ad hoc (CALEC). | Interpersonal:  physical abuse, sexual abuse, parental neglect, parental emotional abuse, exposure to domestic violence, and other stressful events. | Rey Complex Figure Test (Meyers & Meyers, 1995); California Verbal Learning Test (Delis et al., 1987). | Exposed to abuse/neglect offspring had lower cognitive performance (MANCOVA; Wilks’ λ= 0.63, *F*_5,57_ = 6.74, *p* < 0.001) than nonexposed offspring in visual episodic memory (*p*= 0.009) but not in verbal memory, after controlling for socioeconomic status, level of other stressful events, age, sex, Intelligence Quotient (IQ), substance abuse, and non- psychotic DSM diagnoses. | 6 |
| Chiasson et al., 2022 | Canada: Ottawa | Cross-sectional quantitative study | Both Healthy and Clinical | N=26  *M*_age_= 43.2±10.9  CSA^f^ with PTSD^a^ (N=6)  CSA without PTSD (N=9)  Control group (N=11): *M*_age_=35.3±8.9  26 M | Childhood Trauma Questionnaire (CTQ; Bernstein & Fink, 1998); Sexual Victimization Survey (SVS; Finkelhor, 1979); Structured Clinical Interview for Diagnostic and Statistical Manual of Mental Disorders (SCID-5; First et al., 2015). | Interpersonal:  physical abuse, emotional abuse, sexual abuse, physical neglect, and emotional neglect. | Script-driven imagery task ad hoc, Responses to Script-Driven Imagery Scale. (RSDI; Hopper et al., 2007). | CSA participants with (Cluster size 32729, p(FWE-c)=<.001) and without posttraumatic stress disorder (Cluster size 6,520, p(FWE-c)=<.002) had significantly less activity than control participants in the posterior cortices during re-experiencing their traumatic AM, after controlling for age and childhood physical abuse. | 8 |
| Crane et al., 2014 | UK | Longitudinal quantitative study | Healthy | N=5792  13 years old  3297 F, 2495 M | Two event-exposure checklists ad hoc (adapted from Barnett et al., 1983; Brown & Harris, 1978; Coddington, 1972). | Interpersonal:  sexual abuse, being taken into care, parent's death, sibling's death, mother physically cruel, mother’s partner physically cruel, mother very ill, family homeless, mother emotionally cruel and mother’s partner emotionally cruel. | Autobiographical Memory Test (AMT; Williams & Broadbent, 1986). | The results indicated that those who experienced a severe trauma in middle childhood were more likely (60%) to have low specificity of memory at age 13 (OR= 1.61, 95% CI= 1.06-2.47, p= 0.017), after controlling for socio-demographic characteristics, diagnoses of depression, and child depressive symptoms. Therefore, trauma in middle childhood was associated with early adolescent OGM^d^. | 6 |
| D’Amico et al., 2022 | USA | Quantitative study on secondary data | Healthy | N=1541  *M*_age_=53,4±12,4  822 F, 719 M | The Childhood Trauma Questionnaire (CTQ; Bernstein et al., 2003). | Interpersonal:  physical abuse, sexual abuse, emotional abuse, physical neglect, and emotional neglect. | Rey Auditory-Verbal Learning Test included in The Brief Test of Adult Cognition by Telephone (Tun & Lachman, 2006). | Early life adversity may become biologically embedded over time, through the allostatic load, to negatively impact global cognition (β= − 0.01, 95% BCa CI [− 0.01, − 0.001], R2= .27, F(9, 1173)= 48.7, p < .001) and executive functions (β = − 0.01, 95% BCa CI [− 0.01, − 0.001], R2= .25, F(9, 1173)= 44.24, p < .001) in later adulthood; such an effect did not involve episodic memory (β = − 0.004, 95% BCa CI [− 0.01,.0002], R2= .11, F(9, 1169)= 16.85, p < .001), after controlling for age, education, race, lifestyle behaviours, and current depression. | 7 |
| Dawson & Bryant, 2016 | Indonesia: Aceh | Cross-sectional quantitative study | Healthy | N=110  *M*_age_=10.43±1.38  65 F, 45 M | Children’s Revised Impact of Event Scale-13 (CRIES 13; Perrin et al., 2005). | Non-Interpersonal: exposure to tsunami. | Questions ad hoc. | There were significant gender differences in the reconstruction of the trauma memory: girls were more likely to directly recall the tsunami, and boys were more likely to rely on stories from others (χ^2^= 19.08, p < .0001) and to adopt an observer perspective (χ^2^= 15.45, p < .0001). | 4 |
| Ding & He, 2021 | China | Quantitative study on secondary data | Healthy | N=23807  *M*_age_=59.00±10.21  12277 F, 11530 M | China Health and Retirement Longitudinal Study (CHARLS) Life History Survey (Zhao et al., 2014). | Interpersonal: childhood socioeconomic disadvantages, parental-involved trauma, maladaptive parental trauma, and other trauma. | Immediate and delayed word recall test included in CHARLS Survey (Zhao et al., 2014). | Childhood socioeconomic disadvantages (Model 5: − 0.15, 95% CI (− 0.17, − 0.13), p < 0.001), parental-involved trauma (Model 5: − 0.07, 95% CI (− 0.11, − 0.04), p < 0.001), and maladaptive parental trauma (Model 5: -0.09 95% CI (− 0.21,− 0.11), p < 0.001) were associated with a lower score of late-life episodic memory, after controlling for age, sex, survey year, and urban-rural residence. | 6 |
| Feurer et al., 2018 | USA | Longitudinal quantitative study | Healthy | N=502  Mothers group (N=251): *M*_age_=40.38±6.80  251 F  Children group (N=251): *M*_age_=11.40±1.93  51.4% F, 48.6% M | UCLA Life Stress Interview for Children (LSI-C; Adrian & Hammen, 1993). | Miscellaneous (interpersonal and non-interpersonal):  events such as a fight with a friend, failing an exam and a death in the family. | Autobiographical Memory Test (AMT; Williams & Broadbent, 1986). | Elevated levels of stressful life events predicted a decrease of children’s AM specificity for positive (r _effect size_ =- .13) but not negative cues, after controlling for maternal MDD history. | 7 |
| Fishere & Habermas, 2023 | Egypt: Cairo | Cross-sectional quantitative study | Healthy | N=171  *M*_age_=20.46±1.87  Maltreated group (N= 81)  Control group (N=89)  128 F, 43 M | Childhood Experience of Care and Abuse Questionnaire (CECA.Q; Bifulco et al., 2005); Checklist PCL-5 (Weathers et al., 2013). | Interpersonal: antipathy, physical abuse, sexual abuse, and neglect. | Narrative interviews ad hoc. | Narratives of childhood memories by the maltreated group contained fewer emotional words than the control group (F(3,167) = 3.69, p= .013, η^2^= .062). | 7 |
| Fohn et al., 2017 | Belgium | Cross-sectional quantitative study | Healthy | N=26  *M*_age_=73.9±2.8  12 F, 14 M | French version of Impact of Event Scale-Revised (IES-R; Brunet et al., 2003). | Non-Interpersonal: events during and after the war. | Interview ad hoc. | Remembered danger/fear correlated negatively with global evaluations (−.40, p .05) and positively with length (F(1, 22)=5.00, p=.036, η^2^=.19) and dramatic speech (F(1, 22) = 16.15, p = .001, η^2^= .421), after controlling for scariness and danger. | 3 |
| Goldfarb et al., 2019 | USA | Longitudinal quantitative study | Healthy | N=30  Time 1: *M*_age_=8.37±3.61  Time 2: *M*_age_=27.80±3.55  20 F, 10 M | Trauma Symptom Checklist (TSC; Elliot & Briere, 1992), Posttraumatic Diagnostic Scale (PDS; Foa et al., 1997). | Non-Interpersonal: documented medical examination that included genital contact performed to ascertain sexual abuse. | Memory interview ad hoc. | Being older at Time 1 was associated with a greater likelihood of accurately reporting genital contact at Time 2 (r= .40, p= .027). CSA (R^2^= .467, F(5, 21)= 3.66, p = .016, R^2^Δ= .15, bs ≥ .72, SEs ≥.32, βs ≥ .58, ts ≥ 2.24, ps ≤ .038 (ns = 22)) and greater depression (R^2^= .36, F(4, 22) = 3.08, p= .037, R^2^Δ= .18, b= .03, SE= .01, β= .47, t(22)= 2.50, p = .021) in adulthood predicted greater accuracy in reporting of childhood genital touch experienced during the medical exam. Compared with females, males were less likely to report genital contact and were more likely to make omission errors (r= −.44, p= .016), after controlling for age at Time 1. | 5 |
| Goldfarb et al., 2023 | USA | Cross-sectional quantitative study | Healthy | N= 115  *M*_age_=28.31  77 F, 38 M | PTSD Checklist for DSM-5 (PCL-5; Weathers et al., 2013), and Trauma Symptom Checklist (TSC; Elliott & Briere, 1992). | Interpersonal: childhood abuse. | A standard forensic interview (Federal Law Enforcement Training Center Five-Step interview; FLETC) or Cognitive Interview with mental reinstatement (CI-mental) or Cognitive Interview with mental and physical-context reinstatement (CI-context; Fisher & Geiselman, 2018). | Time erodes the ability to remember childhood autobiographical events, particularly for peripheral details. Instead, for traumatic or sensitive memories, some core central facts often remain, sometimes even decades later, at least in adults with histories of suspected maltreatment (F(1, 7)= 15.42, p= .006, η_p_^2^= .69), after controlling for Time 1 age and PTSD Checklist for DSM-5 scores. | 6 |
| Griffith et al., 2016 | Belgium: Leuven | Cross-sectional quantitative study | Clinical | MDD^c^ sample  N=77  *M*_age_=44.7±11.9  45 F, 32 M | The short version of Structured Trauma Interview (STI; Draijer, 2003). | Interpersonal:  physical violence, sexual violence, parental loss, and parental dysfunction. | Dutch version of Autobiographical Memory Test (AMT; Williams and Broadbent 1986). | Childhood physical abuse was related to the recall of fewer spe-cific memories (B= -1.98, SE(B)= .85, β= -.27, ΔR^2^= .09, p<.05), while no association emerged between the AM and childhood sexual trauma (B= 1.43, SE(B)=. 86, β = .21), after controlling for level of depressive symptoms, diagnosis of comorbid disorders, PTSD, and age. | 6 |
| Hakamata et al., 2021 | Tokyo: Japan | Cross-sectional quantitative study | Healthy | N=100  *M*_age_=27.6±11.6  55 F, 45 M | The Childhood Trauma Questionnaire (CTQ; Bernstein & Fink, 1998). | Interpersonal:  physical abuse, physical neglect, emotional abuse, emotional neglect, and sexual abuse. | Autobiographical Memory Test (AMT; Williams & Broadbent, 1986). | More severe childhood trauma was associated with greater semantic-associate memory (r= 0.29, ^FDR-corrected^p= 0.02), one OGM, such that individuals with more severe trauma more recalled the semantic content, but not specific contextual information of an experienced event. In contrast, trauma was not correlated with the other OGM types (i.e., categoric (r= −0.09, ^FDR-corrected^p= 0.65) and extended memory (r= 0.09, ^FDR-corrected^p= 0.65)), after controlling for age, sex, and depression, any potential demographic, and physical condition. | 5 |
| Harris et al., 2016 | USA | Cross-sectional quantitative study | Healthy | N=93  CSA group (N=48): *M*_age_=18.22  Control group (N=45): *M*_age_=17.97  81 F, 12 M | Posttraumatic Diagnostic Scale (PDS; Foa et al., 1997) or Child PTSD Symptom Scale (CPSS; Foa et al., 2001); Trauma Symptom Checklist-40 (TSC-40; Briere & Runtz, 1989) or Trauma Symptom Checklist for Children (TSC-C; Briere, 1996); Trauma Assessment for Adults (TAA; Resnick et al., 1993) or Traumatic Events Screening Inventory for Children (TESI-C; Ford & Rogers, 1997); Negative Experiences Questionnaire (short version of the Sexual Assault Profile; Goodman et al., 1992); Childhood Trauma Questionnaire (CTQ; Bernstein et al., 1994). | Interpersonal:  sexual abuse, physical abuse, emotional abuse, physical neglect, emotional neglect, and lifetime trauma. | Autobiographical Memory Interview (AMI; Kopelman, 1994; Kopelman et al., 1989). | Reduced AM specificity was associated to emotional and physical abuse in childhood (βs ≤ \|−.21\|, ps ≤ .05; overall models, Fs_(7, 83)_ ≥4.34, ps < .001), but not with CSA (F_(6, 32)_= 2.25, p= .06, β= .03), after controlling for age, vocabulary, trauma-related psychopathology, coping, and maltreatment variables. | 7 |
| Hawkins et al., 2020 | USA | Cross‐sectional quantitative study | Healthy | Adults with excess adiposity  N=95  *M*_age_=45.6±11.8  72 F, 23 M | Adverse Childhood Experiences Survey (ACEs Survey; Felitti et al., 1998). | Interpersonal: emotional abuse, physical abuse, sexual abuse, emotional neglect, physical neglect, domestic violence, parental separation/divorce, familial mental illness, substance use, and incarceration. | Picture Sequence Memory included in NIH Toolbox Cognition Battery (NIHTB‐CB; Weintraub et al., 2013). | ACEs^e^ were associated with poorer episodic memory (F_1,88_= 6.40, P= .013, partial η^2^= .07), after controlling for body mass index, age, sex, race, and education. | 6 |
| Hitchcock et al., 2014 | Australia | Longitudinal quantitative study | Healthy | N=50  *M*_age_=11.90±3.31  10 F, 40 M | Adaptation of the Cambridge Life Development Measure (Goodyer et al., 1997) and Child Post-Traumatic Stress Scale (CPSS; Foa et al., 2001). | Non-Interpersonal: motor vehicle accidents, extreme sports accidents, incidents with dangerous equipment, and falls. | Autobiographical Memory Test (AMT; Williams & Broadbent, 1986). | A significant negative relationship between OGM and PTSD symptoms emerged 6 months after trauma (b= -2.59, SE_b_= 1.10, 95% CI [-4.70, -0.47], χ^2^(2)= 9.07, p= .01). OGM was protective against PTSD: OGM may have aided participants in regulating the negative effects produced by their trauma memory. | 5 |
| Huntjens et al., 2014 | Netherlands and Belgium | Cross-sectional quantitative study | Both Healthy and Clinical | N=93  Dissociative identity disorder (DID) group (N=12): *M*_age_=41  PTSD group (N=26): *M*_age_=41  Control group (N=29): *M*_age_=39  DID simulators group (N=26): *M*_age_=46  93 F | Symptom Scale Self-Report version (PSS-SR; Foa et al., 1993); Acceptance and Action Questionnaire-Trauma Specific (AAQ-TS; Land, 2011); Posttraumatic Avoidance Behavior Questionnaire (PABQ; van Minnen & Hagenaars, 2010); Traumatic Experiences Checklist (TEC; Nijenhuis et al., 2002). | Interpersonal: childhood emotional trauma, childhood physical abuse and childhood sexual trauma | Autobiographical Memory Task (AMT; Williams & Broadbent, 1986). | Both PTSD (p= .004) and DID (p= .037) patients showed OGM compared to controls (F(2, 62)= 5.18, p= .008, partial η^2^= .14), after controlling for depression. | 9 |
| Jiang et al., 2020 | China | Cross-sectional quantitative study | Both Healthy and Clinical | N=356  Depression group (N=180): *M*_age_=29.5±8  Control group (N=176): *M*_age_=28.4±8.8  186 F, 170 M | The Chinese version of the Childhood Trauma Questionnaire (CTQ-SF; Fu & Yao, 2005). | Interpersonal:  sexual abuse, physical abuse, emotional abuse, emotional neglect, and physical neglect. | OGM questionnaire (OGMQ; Keyu, 2016). | Childhood trauma had an indirect effect on the current suicidal ideation through OGM in depressed group (β= 0.062, 95% CI: 0.023–0.117; P= 0.002), but not in control group (β= 0.002; 95% CI: − 0.005–0.017; P= 0.337). | 9 |
| Kaczmarczyk et al., 2018 | Germany: Berlin | Cross-sectional quantitative study | Both Healthy and Clinical | N=143  MDD group (N=68): *M*_age_=37.4±9.3  54.4% F, 45.6% M  Control group (N=75): *M*_age_=35.1±9.2  65.3% F, 34.7% M | Childhood Trauma Questionnaire (CTQ; Bernstein and Fink, 1998; Wingenfeld et al., 2010). | Interpersonal:  sexual abuse, physical abuse, emotional abuse, physical neglect, and emotional neglect. | Modified version of Autobiographic Memory Test (AMT; Buss et al., 2004). | Higher CTQ scores (β= −0.298; t= −3.142; p= 0.002) but not MDD were associated with less specific AM (F(5;137)= 4.21, p < 0.01) with R^2^= 0.13 (adjusted R^2^= 0.10), after controlling for CTQ sum score. | 9 |
| Kangaslampi, 2023 | Finland: Tampere | Cross-sectional quantitative study | Healthy | N=166  *M*_age_=10.87±0.79  84 F, 81 M, 1 Missing | A questionnaire ad hoc assessed trauma as the content of earliest memories and AM. | Miscellaneous (interpersonal and non-interpersonal) | A questionnaire ad hoc. | No differences between earliest and other autobiographical memories were noted in specificity (McNemar’s χ^2^(1)= 0.19, p= .665), trauma and accident-related content (χ^2^(1)= 0.30, p= .584), or emotional content (positive emotions: χ^2^(1)= 2.37, p= .124; negative emotions: χ^2^(1)= 0.378, p= .540). | 4 |
| Kaynar & Er, 2015 | Turkey | Study 1: Cross-sectional quantitative  Study 2:  Cross-sectional quantitative | Healthy | Study 1: N=198  *M*_age_=21.71±2.47  129 F, 69 M  Study 2: N=46  *M*_age_=22.03±2.33  28 F, 18 M | Study 1: Adapted version of Childhood Trauma Questionnaire (CTQ; Aslan & Alparslan, 1999); Adapted version of Impact of Event Scale-Revised (IES-R; Çorapçıoğlu et al., 2006).  Study 2: Adapted version of Impact of Event Scale-Revised (IES-R; Çorapçıoğlu et al., 2006); Form Autobiographical Event Content (FAEC) ad hoc assessed duration and source of abuse. | Interpersonal: emotional neglect, emotional abuse, sexual abuse, physical neglect, and physical abuse | Study 1: Autobiographical Memory Characteristics Inventory (AMCI; Er, 2005); Brief Autobiographical Memory Effects Inventory (BAMEI; Er & Boyraz, 2012).  Study 2: Autobiographical Memory Characteristics Inventory (AMCI; Er, 2005). | The participants with higher levels of childhood trauma recalled more detailed negative memories (t_101_= 2.42, p= .034, d= .57) and less detailed positive (t_101_= 2.40, p=.004, d= .56) and recent past (t_101_= 2.71, p= .007, d= .50) memories. Childhood trauma experiences predicted OGM (R^2^= .09, F_1,45_= 15.22, β= .24, t_46_= 2.55, p < .001). | 5 |
| Lawson et al., 2021 | USA:  St. Joseph County | Longitudinal quantitative study | Healthy | N=104:  Maltreated children group (N=52): *M*_age_=4.59±1.06  32 F, 20 M  Mothers group (N=52) | Forensic interviews. | Interpersonal:  sexual abuse, physical abuse, neglect, and emotional maltreatment. | Autobiographical Emotional Events Dialogue procedure (Koren-Karie et al., 2003). | Maternal elaboration was positively associated with children’s autobiographical memory in forensic reports when mothers are autonomy-supportive during reminiscing (b= 0.30, p= .01).  In addition, older children provided more unique details than younger children (R^2^Δ= .02, R^2^= .81, b= 0.79, SE= .26, β= .24, p= .004), and females also provided more unique details than males (b= -1.13, SE= .55, β= -.15, p= .046), after controlling for variability in interviewers’ questions and statements, children’s memory during reminiscing, age, and gender. | 6 |
| Lin et al., 2022 | China | Quantitative study on secondary data | Healthy | N=6466  *M*_age_=57.2±8.3  3165 F, 3301 M | Interviews ad hoc. | Interpersonal:  physical abuse, household substance abuse, domestic violence, unsafe neighborhoods, bullying, emotional neglect, household mental illness, incarcerated household member, parental separation or divorce, and parental death. | Questionnaire ad hoc adapted from Telephone Interview for Cognitive Status (Fong et al., 2009). | A global cognitive decline over time (episodic memory and executive function) was found after two or more deprivation-related ACEs (β= −0.035, 95% CI: [−0.050 to −0.019] for global cognition; β= −0.047, 95% CI: [−0.068 to −0.025] for episodic memory; β= −0.019, 95% CI: [−0.034 to −0.004] for executive  function), after controlling for demographic characteristics, ethnicity, childhood socioeconomic status, threat-related ACEs, and deprivation-related ACEs. | 5 |
| McCrory et al., 2017 | UK: London | Cross-sectional quantitative study | Healthy | N=67  Maltreatment group (N=34): *M*_age_=12.53±1.6  Control group (N=33): *M*_age_=12.66±1.29  36 F, 31 M | Childhood Trauma Questionnaire (CTQ; Bernstein et al., 2003). | Interpersonal:  physical abuse, sexual abuse, emotional abuse, emotional neglect, and physical neglect. | Two subtests of the CogState battery (Maruff et al., 2013); Autobiographical Memory Test (AMT; Williams & Broadbent, 1986). | The maltreatment group generated more OGM across conditions (t(55)= -2.46, P= 0.017, 95% CI: -0.22 to -0.02). | 6 |
| McKinnon et al., 2017 | Australia | Study 1:  Cross-sectional quantitative  Study 2:  Longitudinal quantitative | Healthy | Study 1: N=36  *M*_age_=10.41±3.15  11 F, 25 M  Study 2: N=57  *M*_age_=11.81±2.13  Sex not specified | Study 1: Children's Acute Stress Questionnaire (CASQ; Kassam-Adams, 2006) and Child PTSD Symptom Scale (CPSS; Foa et al., 2001).  Study 2: Child PTSD Symptom Scale (CPSS; Foa et al., 2001). | Non-Interpersonal: stressful orthopaedic procedure (Study 1), accidental injury (Study 2). | Study 1: Interview ad hoc.  Study 2: Interview ad hoc. | In both studies participants recalled their stressful experiences accurately regardless of post traumatic stress severity (Study 1: r= 0.17, p= 0.32, 95% CI: [-0.17, 0.47]; Study 2: r= 0.08, p= 0.55, 95% CI: [-0.18, 0.33]), after controlling fear, pain, intelligence, mood, demographic factors, site differences, age, the presence of clowns during procedures, PTSD symptoms, injury severity, family history, and gender. | 4 |
| Neshat Doost et al., 2014 | Iran | Cross-sectional quantitative study | Healthy | N=103  Bereaved group (N=70): *M*_age_=14.87±1.83  Control group (N=33): *M*_age_=14.91±2.05  49 F, 54 M | Persian version of the Impact of Event Scale (IES; Horowitz et al., 1979). The trauma was already known. | Interpersonal:  father's death due to war. | Autobiographical Memory Task (AMT; Williams & Broadbent, 1986). | The bereaved group had impaired AM specificity (F(1,101)= 27.15, p < .001, η^2^_p_= .21) and greater extended (F(1, 101)= 12.61, p= .001, η^2^_p_= .11) and categoric (F(1,101)= 12.14, p= .001, η^2^_p_= .11) memories than the control group, after controlling for depression. | 8 |
| Pacheco & Scheeringa, 2022 | USA:  New Orleans | Cross-sectional quantitative study | Healthy | N=257  Trauma group (N=216): *M*_age_=5.1±1.1  Control group (N=41): *M*_age_=4.7±0.9  100 F, 157 M | Modified measure ad hoc of Preschool Age Psychiatric Assessment (PAPA). | Miscellaneous (interpersonal and non-interpersonal):  motor vehicle accidents, accidental injuries, witnessed single incidents of relatives murdered, assaulted, or being severely injured, witnessed domestic violence and Hurricane Katrina-related trauma events. | Interviews ad hoc. | Young children who experienced repeated trauma events or Hurricane Katrina recalled trauma details less accurately than those who experienced single-event trauma (F= 7.18, p= .001). No difference was found in the accuracy of recall of the traumatic event in the trauma-exposed group compared to the accuracy of recall of the stressful event in the non-trauma-exposed control group (F= 0.00, p= .95), after controlling for age, amount of naturalistic family rehearsal, demographics, rehearsal, duration between trauma and assessments, and misleading question scores. | 7 |
| Parlar et al., 2016 | Canada: Hamilton | Cross-sectional quantitative study | Both Healthy and Clinical | N=41  MDD with trauma group (N=21): *M*_age_=41.3±14.5  Control group (N=20): *M*_age_=36.5±13.4  21 F, 20 M | Childhood Trauma Questionnaire (CTQ; Bernstein et al., 2003); Clinician-Administered PTSD Scale (CAPS; Blake et al., 1995). | Miscellaneous (interpersonal and non-interpersonal): emotional abuse, physical abuse, sexual abuse, emotional neglect, and physical neglect, single-blow, accidental trauma (e.g., car accident). | A modified version of Crovitz’s cue-word test (Addis et al., 2009; Baddeley & Wilson 1986). | Levels of episodic simulation were unrelated to symptom severity, childhood trauma severity, and number of depressive episodes. | 8 |
| Peltonen et al., 2017 | Palestine: Gaza Strip | Longitudinal quantitative study | Healthy | N=240  *M*_age_=11.35±0.57  49.4% F, 50.6% M | Checklist of 28 traumatic events ad hoc and Children’s Revised Impact of Events Scale (CRIES; Smith et al., 2003). | Non-Interpersonal: war trauma. | AM enquiry ad hoc. | Children exposed to severe war trauma did not show more generalized memories (B= .145, SE(B)= .052, χ^2^= 7.835, OR= 1.156, 95% CI: 1.044–1.280, p= .005). | 7 |
| Risløv Staugaard et al., 2017 | Denmark: Odder | Cross-sectional  quantitative study | Healthy | N=136  *M*_age_=17.3  82 F, 54 M | Posttraumatic Checklist-Civilian Version (PCL-C; Karstoft et al., 2014). | Non-Interpersonal: dental treatment. | Autobiographical Memory Questionnaire (AMQ; Rubin & Berntsen, 2009). | Negative memories of dental treatment were associated with both dental fear and PTSD symptoms: specifically, some features of negative memories, such as vividness and clarity (0.37, 95% CI: [0.17, 0. 54], P<0.05), were associated only with dental fear, while others, such as spontaneous recall (0.30, 95% CI: [0.09, 0.49], P<0.05 for dental fear; 0.26, 95% CI: [0.05, 0.45], P<0.05 for PTSD symptoms), also with PTSD symptoms. | 5 |
| Saleh et al., 2017 | USA: Nashville, Durham | Cross-sectional quantitative study | Both Healthy and Clinical | N=129  MDD group (N=64): *M*_age_=35.1±8.9  Control group (N=65): *M*_age_=29.7±9.2  82 F, 47 M | A modified version of the self-report Early Life Stress Questionnaire (ELSQ), developed from the Child Abuse and Trauma Scale (Sanders & Becker-Lausen, 1995). | Miscellaneous (interpersonal and non-interpersonal): emotional trauma, physical abuse, sexual abuse, domestic violence, severe family conflict, neglect, divorce, separated, death in the family, major illness in the family, a fire destroyed home, war, natural disaster, major personal illness, hospitalization/surgery, bullied, premature birth, adoption, and other events. | Benton Visual Retention Test (Benton, 1991); Rey's Verbal Learning Test (Rey, 1964); two subtests of Wechsler Memory Scale (Wechsler, 2009): Logical Memory 1 and 2. | Childhood traumatic experiences were not associated with performance in episodic memory, regardless of depression condition (F= 2.82 (1,125), p= 0.0954), after controlling for age, sex, education, medical morbidity, intracranial volume, and diagnosis of MDD. | 9 |
| Salomão et al., 2021 | Portugal | Cross-sectional quantitative study | Healthy | N=56  *M*_age_=9.53±1.61  39.3% F, 60.7% M | Children were referred to Child Protective Services for exposure to domestic violence, neglect, abuse, and disruptive behavior. | Interpersonal: domestic violence, neglect, abuse. | Adapted version of the Episodic Thinking Interview (Coughlin et al., 2014). | Children exposed to domestic violence, neglect and abuse had significantly lower episodic memory performance, compared to children referred for disruptive behavior (F= 8.80, df= (2,52), η^2^_p_= .25, Power= .96, p < .001), after controlling for age. | 6 |
| Staniloiu et al., 2018 | Not specified | Longitudinal qualitative study | Clinical | Sample with a diagnosis of dissociative amnesia  N=28  *M*_age_=35.6  7 F, 21 M | No direct measurement of childhood trauma. | Miscellaneous (interpersonal and non-interpersonal):  car accidents, previous history of sexual abuse, professional failures, stressful events. | Modified version of the Autobiographical Memory Interview (Kopelman et al., 1990). | Results revealed that 25 out of 28 patients suffered different types of trauma in childhood, suggesting “a mechanism of incubation of trauma or kindling desensitization”. | 3 |
| Thomson & Jaque, 2022 | USA | Longitudinal quantitative study | Healthy | N=130  *M*_age_=27.41±10.06  76 F, 54 M | Adverse Childhood Experiences Questionnaire (ACE; Felitti et al., 1998). | Interpersonal:  abuse, neglect, and household dysfunctions. | Amnestic Scale of Dissociative Experience Scale – II (DES; Bernstein & Putnam, 1986). | Retrospective reporting of ACEs was stable over time (r= .896, p < .01), after controlling for sex, age, and ethnicity. | 7 |
| Tian et al., 2018 | China | Cross-sectional quantitative study | Healthy | N=93  Earthquake trauma group (N=47): *M*_age_=14.77±0.56  Control group (N=46): *M*_age_=14.60±0.39  52 F, 41 M | Earthquake-Related Experiences Questionnaire (EREQ) ad hoc. | Non-Interpersonal: earthquake-related trauma. | Autobiographical Memory Test (AMT; Williams & Broadbent, 1986; Roberts & Carlos, 2006). | Adolescents exposed to earthquake trauma reported more depression (t(91)= 2.38, p= 0.02, d= 0.58) and more OGM (t(91)= 2.66, p= 0.009, d= 0.48). | 8 |
| Vallet et al., 2017 | Spain: Madrid | Cross-sectional quantitative study | Healthy | N=196  Adults group (N=104): *M*_age_=39.41 at time of terrorist attacks  Youths group (N=92): *M*_age_=9.60 at time of terrorist attacks  133 F, 63 M  Measurements were 12 years later. | No measurement of trauma, all participants were living in Madrid at the time of the terroristic attacks. | Non-Interpersonal: exposure to terroristic attacks. | Phenomenological Questionnaire for Autobiographical Memory (Manzanero & López, 2007). | There were differences between the two groups in the quality and accessibility of memory infor-mation. Young memories of the attacks had less quality memory, such as less definition (t= -7.76, df= 166, p= .000, d’= 1.11), less vividness (t= -6.81, df= 194, p= .000, d’= 0.97) and fewer details (t= -6.40, df= 194, p= .000, d’= 0.92), and were less complex (t= -3.54, df= 193, p= .000, d’= 0.51) and understandable (t= -2.87, df= 194, p= .005, d’= 0.41). The older adults appeared to be more reliable. The young group has obtained a higher score in doubts about their own memory (t= 4.87, df= 194, p= .000, d’= 0.70). | 6 |
| Varnaseri et al., 2016 | UK | Cross-sectional quantitative study | Healthy | N=34  *M*_age_=40.32±12.53  34 M | Childhood Trauma Questionnaire (CTQ; Bernstein & Fink, 1998). | Interpersonal: emotional abuse, physical abuse, sexual abuse, emotional neglect, and physical neglect. | Autobiographical Memory Test (AMT; Williams & Broadbent, 1986). | The incidence of childhood emotional abuse, physical abuse, sexual abuse, emotional neglect, and physical neglect was associated with impaired AM retrieval (b=0.45, 95% CI: [0.028, 1.165). | 4 |
| Viard et al., 2019 | France: Caen | Cross-sectional quantitative study | Both Heathy and Clinical | N=38  PTSD group (N=14): *M*_age_=15.73±1.51  Control group (N=24): *M*_age_=15.99±1.72  25 F, 13 M | Structured Clinical Interview-Clinician Version (SCID-CV; First et al., 2002; Lobbestael et al., 2011); French version of the Impact of the Event Scale-Revised (IES-R; Brunet et al., 2003; Weiss & Marmar, 1997). | Miscellaneous (interpersonal and non-interpersonal):  sexual abuse, accident, loss of loved one, witness of suicide. | The French version of the Children's Memory Scale (CMS; Cohen, 1997). | PTSD patients had significantly lower performances for both immediate (t= 2.13, p < .040) and delayed (t= 2.19, p= .035) recall compared to controls, after controlling for the time courses from white matter, cerebrospinal fluid, their derivatives, the six movements parameters generated from realignment of head motion, age, and sex. | 8 |
| Wang et al., 2016 | China | Cross-sectional quantitative study | Both Healthy and Clinical | N=137  Mild cognitive  impairment (MCI) group (N=76): *M*_age_=73.26±5.59  Control group (N=61): *M*_age_=70.48±5.05  77 F, 60 M | Childhood Trauma Questionnaire – Brief Version (CTQ-RF; Fu et al., 2005). | Interpersonal: emotional neglect, emotional abuse, sexual abuse, physical neglect, and physical abuse. | Wechsler Memory Scale (Wechsler, 2009). | Early childhood physical neglect had a major role in the decrease in learning (r= -0.09, p= 0.28) and memorizing capability, particularly in the impairment of episodic memory (r= -0.26, p= 0.00). | 7 |
| Weems et al., 2014 | USA:  New Orleans | Longitudinal quantitative study | Healthy | Study 1:  N=94  *M*_age_=14.2  58% F, 42% M  Study 2:  N=141  *M*_age_=11.5  47% F, 53% M | Posttraumatic Stress Reaction Index for Children (PTSD-RI; La Greca et al., 1996; La Greca et al., 1998; Weems et al., 2010) and short form of the Life Events Checklist (LEC; Johnson & McCutcheon, 1980). | Non-Interpersonal: exposure to Hurricane Katrina and Hurricane Gustav. | Survey ad hoc; Questions ad hoc. | Study 1: low negative Gustav exposure was associated with decreases in reports of Katrina events, while high negative Gustav exposure was associated with more stability in reports (p < .001 for effect of Gustav exposure).  Study 2: A high exposition to another similar traumatic (Gustave) event leads to a reconsolidation of the memories, but a low post-reactivation of a similar traumatic event slowed down the initial memory reconsolidation (p < .01 for effect of Gustav exposure), that is few memories of the first traumatic event (Katrina), after controlling for initial levels of PTSD symptoms and level of Gustav exposure. | 6 |
| Wittekind et al., 2016 | Germany | Cross-sectional quantitative study | Healthy | N=65  Offspring of elderly traumatized without PTSD group (N=22): *M*_age_=43.6±4.8  Offspring of elderly traumatized with PTSD group (N=20): *M*_age_=43.3±7.5  Offspring of elderly non-traumatized group (N=23): *M*_age_=43.0±5.3  44 F, 21 M | No measurement of trauma in offspring, parents had war trauma. | Non-Interpersonal: war trauma. | Adapted version of Autobiographical Memory Task (AMT; Williams & Broadbent, 1986). | No differences in AM specificity between groups (F(2,62)= 0.33, p= 0.72); therefore, no evidence for a transgenerational effect of trauma and psychopathology in parents on AM specificity in offspring emerged. | 8 |
| Wittekind et al., 2017 | Germany | Cross-sectional quantitative study | Both Healthy and Clinical | N=67  Traumatized without PTSD group (N=25): *M*_age_=72.84±1.86  Traumatized with PTSD group (N=19): *M*_age_=72.58±2.22  Control group (N=23): *M*_age_=73.78±2.92  50 F, 17 M | Structured Clinical Interview for PTSD (SCID; Wittchen et al., 1997) and Post-traumatic Diagnostic Scale (PDS; Foa et al., 1997). | Non-Interpersonal: war trauma. | Adapted version of Autobiographical Memory Task (AMT; Williams & Broadbent, 1986). | No difference between groups in their ability to recall specific memories of their past (F(2,63)= 1.32, p= 0.27, η2p= 0.04). PTSD is not associated with AM impairment in the elderly (\|r\| < 0.31, ps > 0.20). | 9 |
| Wolf & Nochajski, 2022 | USA | Cross-sectional quantitative study | Healthy | N=297  Age ranged from 18 to 73 years  258 F, 39 M | CSA Subscale of the Computer Assisted Maltreatment Inventory (CAMI; DiLillo et al., 2010); Brief Betrayal Trauma Survey (BBTS; Goldberg & Freyd, 2006). | Interpersonal:  sexual abuse and betrayal trauma. | Memory questions adopted from The Childhood Sexual Abuse Inventory-Revised (CSAI; Cook & Stock, 1995); Autobiographical Memory Loss Questions adopted from ACES instrument (Edwards et al., 2001). | CSA dissociative amnesia was related to autobiographical memory loss (b= 0.534, SE= 0.153, p= .0001, CI: 0.252-0.014) ; in addition, earlier age at the onset of abuse was a risk factor for both dissociative amnesia and autobiographical memory loss (b= -0.047, p= .035). | 5 |
| Zhang et al., 2023 | China | Quantitative study on secondary data | Healthy | N=14484  *M*_age_=60.7±9.5  7559 F, 6925 M | A questionnaire ad hoc. | Interpersonal:  ACEs (child maltreatment, exposure to violence, parent/sibling death or disability, and parental maladjustment). | A word-recall test adopted from the CHARLS procedure (Li et al., 2017; Rong et al., 2020). | No significant association be- tween accumulated ACES exposure and episodic memory in adulthood was observed (aOR= -0.120, 95% CI: -0.241, 0.001), after controlling for sociodemographic characteristics, behaviours, lifestyles, and chronic conditions. | 5 |
| Zhu & Hakim-Larson, 2022 | Canada: Ontario | Cross-sectional quantitative study | Healthy | N=204  *M*_age_=25.52±8.50  159 F, 40 M, 2 gender non-binary, 1 trans, 2 missing. | No measurement of trauma, all participants experienced one or more child maltreatment. | Interpersonal:  child maltreatment (e.g., sexual, physical, and emotional abuse and neglect). | Questions ad hoc adapted from the Autobiographical Memory Questionnaire (Rubin et al., 2008). | Maltreatment narratives were more coherent than positive event narratives (t(189)= -6.90, p < .001, 95% CI: [-.93, -.52], Cohen's d= .50 for chronology; t(189)= -2.25, p= .026, 95% CI: [-.22, -.01], Cohen's d= .16 for theme coherence), and included more information related to the temporal order, elaboration, and meaning-making of the event, before considering covariates. Maltreatment narratives were more chronologically coherent than positive event narratives (F(1, 208)= 34.20, p < .001, 95% CI: [.40, .81]), and there was no significant difference for theme coherence, after controlling for reading level of narrative, length of narrative, rehearsal of event, and retention interval. | 5 |

Note. a PTSD: Post Traumatic Stress Disorder; b BPD: Borderline Personality Disorder; c MDD: Major Depressive Disorder; d OGM: Overgeneral Memory; e ACEs: Adverse Childhood Experiences; f CSA: Child or Childhood Sexual Abuse; g AM: Autobiographical Memory; h CI: Confidence Interval.
