# Supplementary material for: The relationship between childhood interpersonal and non-interpersonal trauma and autobiographical memory: a systematic review
Source: Front Psychol. 2024 Jan 17;15:1328835. doi: 10.3389/fpsyg.2024.1328835 (PMC10827865; doi:10.3389/fpsyg.2024.1328835)
Supplement: Supplementary file 2 [file Table_2.DOCX]

**Supplementary Materials**

[Glossary of terms 2](#_Toc154165981)

[Literature search and search strategies 3](#_Toc154165982)

[PubMed 3](#_Toc154165983)

[Scopus 3](#_Toc154165984)

[Web of Science 3](#_Toc154165985)

[Full list of excluded studies from eligible studies 5](#_Toc154165986)

[Definition of exposure and outcome variables 7](#_Toc154165987)

[Exposure 7](#_Toc154165988)

[Outcome 7](#_Toc154165989)

[PRISMA 2020 statement and checklist 8](#_Toc154165990)

[PRISMA 2020 for abstracts checklist 13](#_Toc154165991)

[Risk of bias and quality assessment of included studies - Newcastle-Ottawa scale for non-randomised studies 15](#_Toc154165992)

[References 20](#_Toc154165993)

# Glossary of terms

DSM: Diagnostic and Statistical Manual of Mental Disorders

PTSD: Post-Traumatic Stress Disorder

BD: Bipolar Disorder

MDD: Major Depressive Disorder

AM: Autobiographical Memory

OGM: Overgeneral Memory

CaR-FA-X model: CaR (Capture and Rumination); FA (Functional Avoidance); X (Poor Executive Control)

FA: Functional Avoidance

CaR: Capture and Rumination

X: Poor Executive Control

PRISMA: Preferred Reporting Items for Systematic Reviews and Meta-analyses

CTQ: Childhood Trauma Questionnaire

AMT: Autobiographical Memory Test

NOS: Newcastle-Ottawa Scale

DID: Dissociative Identity Disorder

MCI: Mild Cognitive Impairment

BPD: Borderline Personality Disorder

DBTF: Development-Based Trauma Framework

PTG: Post-Traumatic Growth

# Literature search and search strategies

Four reviewers independently collected data from electronic databases (PubMed, Scopus, Web of Science). The search strategy was carried out from January 2023 to March 2023. The keyword combinations used for each electronic database are as follows:

## **PubMed**

“autobiographical memory” AND (“childhood trauma” OR “children trauma” OR “child trauma” OR “developmental trauma”), “autobiographical memory” AND (“childhood PTSD” OR “children PTSD” OR “child PTSD” OR “developmental PTSD”), “autobiographical memory” AND (“childhood post-traumatic stress disorder” OR “children post-traumatic stress disorder” OR “child post-traumatic stress disorder” OR “developmental post-traumatic stress disorder”).

Used filter: 2014-2023.

Records identified: 339.

## **Scopus**

“autobiographical memory” AND (“childhood trauma” OR “children trauma” OR “child trauma” OR “developmental trauma”), “autobiographical memory” AND (“childhood PTSD” OR “children PTSD” OR “child PTSD” OR “developmental PTSD”), “autobiographical memory” AND (“childhood post-traumatic stress disorder” OR “children post-traumatic stress disorder” OR “child post-traumatic stress disorder” OR “developmental post-traumatic stress disorder”).

Used filter: 2014-2023.

Records identified: 186.

## **Web of Science**

“autobiographical memory” AND (“childhood trauma” OR “children trauma” OR “child trauma” OR “developmental trauma”), “autobiographical memory” AND (“childhood PTSD” OR “children PTSD” OR “child PTSD” OR “developmental PTSD”), “autobiographical memory” AND (“childhood post-traumatic stress disorder” OR “children post-traumatic stress disorder” OR “child post-traumatic stress disorder” OR “developmental post-traumatic stress disorder”).

Used filter: 2014-2023.

Records identified: 316.

After the initial research we excluded records screened for abstract and title for the following reasons:

| **Inclusion Criteria for articles** | **Exclusion Criteria for articles** |
| --- | --- |
| They were published in the period from 2014 to 2023 | They were review papers |
| They were written in English | They were studies that commented on other studies |
| They investigated the relationship between AM and childhood interpersonal and non-interpersonal trauma | They were studies that did not involve human beings |
| They included a sample of children, adolescents, or adults who had experienced childhood interpersonal and/or non-interpersonal trauma | They were editorial essay |
|  | They were duplicates in the search of other databases |
|  | They were studies that did not focus on the topic of interest |
|  | They were theoretical articles |
|  | They were not written entirely in English |
|  | The full article was not available |

#

# Full list of excluded studies from eligible studies

| **Authors, Year** | **Title** | **Reason for exclusion** |
| --- | --- | --- |
| Akbarian et al., 2015 | The effectiveness of cognitive behavioral therapy with respect to psychological symptoms and recovering autobiographical memory in patients suffering from post-traumatic stress disorder. | It assessed efficacy of intervention |
| Askelund et al., 2019 | Positive memory specificity is associated with reduced vulnerability to depression. | Different focus |
| Badura Brack et al., 2022 | Neurostructural brain imaging study of trait dissociation in healthy children. | It did not measure autobiographical/episodic memory |
| Bellido-Zanin et al., 2018 | Childhood memories of threatening experiences and submissiveness and its relationship to hallucination proneness and ideas of reference: The mediating role of dissociation. | Different focus |
| Duesenberg et al., 2019 | Psychophysiological stress response and memory in borderline personality disorder. | Different focus |
| Frampton et al., 2018 | The effects of adult depression on the recollection of adverse childhood experiences. | It did not measure autobiographical/episodic memory |
| Guhn et al., 2018 | Affective and cognitive reactivity to mood induction in chronic depression. | Different focus |
| Guo et al., 2023 | Relationship between childhood maltreatment and cognitive function in medication-free patients with major depressive disorder. | It did not measure autobiographical/episodic memory |
| Joss et al., 2020 | Effects of a mindfulness based behavioral intervention for young adults with childhood maltreatment history on hippocampal morphometry: a pilot MRI study with voxel-based morphometry. | It assessed efficacy of intervention |
| Kangaslampi et al., 2015 | Narrative exposure therapy for immigrant children traumatized by war: study protocol for a randomized controlled trial of effectiveness and mechanisms of change. | It assessed efficacy of intervention |
| Kluemper & Dalenberg, 2014 | Is the dissociative adult suggestible? A test of the trauma and fantasy models of dissociation. | Different focus |
| Lebois et al., 2020 | Higher integration scores are associated with facial emotion perception differences in dissociative identity disorder. | Different focus |
| Li et al., 2021 | Hippocampal subfield alterations in pediatric patients with post-traumatic stress disorder. | It did not measure autobiographical/episodic memory |
| Mertens et al., 2022 | Neural correlates of acute post-traumatic dissociation: a functional neuroimaging script-driven imagery study. | Different focus |
| Metz et al., 2019 | Effects of hydrocortisone on autobiographical memory retrieval in patients with posttraumatic stress disorder and borderline personality disorder: the role of childhood trauma. | Different focus |
| Nuttall et al., 2014 | Autobiographical memory specificity among preschool-aged children. | Different focus |
| Patriat et al., 2016 | Default-Mode Network Abnormalities in Pediatric Posttraumatic Stress Disorder. | It did not measure autobiographical/episodic memory |
| Poli et al., 2022 | Randomized Trial on the Effects of a Group EMDR Intervention on Narrative Complexity and Specificity of Autobiographical Memories: A Path Analytic and Supervised Machine-Learning Study. | It assessed efficacy of intervention |
| Vrijsen et al., 2019 | Effect of cognitive bias modification-memory on depressive symptoms and autobiographical memory bias: two independent studies in high-ruminating and dysphoric samples. | It assessed efficacy of intervention |
| Zeev-Wolf et al., 2020 | Cumulative Risk on Oxytocin-Pathway Genes Impairs Default Mode Network Connectivity in Trauma-Exposed Youth. | Different focus |

#

# Definition of exposure and outcome variables

## **Exposure**

Childhood trauma is configured as interpersonal when it is caused by the violence acted by one or more perpetrators known or unknown to the victim (i.e., physical/emotional/sexual abuse and/or physical/emotional neglect, including domestic violence, bullying and violence in institutional settings), occurring before age 18 (Baker et al., 2021; De Bellis and Zisk, 2014; Musicaro et al., 2020; Parker et al., 1979; Teicher and Samson, 2013; Thomas et al., 2021).

Childhood trauma is configured as non-interpersonal when the traumatization is caused by non-human forces (i.e., motor vehicle accidents, natural disasters, diseases) (De Bellis and Zisk, 2014; Maharaj et al., 2022; Musicaro et al, 2020).

We refer to Overall trauma when both interpersonal and non-interpersonal trauma occurred in the same sample.

## **Outcome**

Autobiographical memory can be defined as “The ability to remember personal events is at the heart of what defines an individual as a person with obligations, roles, and commitments in a given society. It enables us to draw lessons from our past and plan our personal future. It helps us to orient and participate in complex social communities. Autobiographical memory is therefore crucial for a sense of identity, continuity, and direction in life.” (Berntsen & Rubin, 2012, p. 1).

# PRISMA 2020 statement and checklist

| **Section and Topic** | **Item #** | **Checklist item** | **Location where item is reported** |
| --- | --- | --- | --- |
| **TITLE** | | |  |
| Title | 1 | Identify the report as a systematic review. | Cover page |
| **ABSTRACT** | | |  |
| Abstract | 2 | See the PRISMA 2020 for Abstracts checklist. | Abstract |
| **INTRODUCTION** | | |  |
| Rationale | 3 | Describe the rationale for the review in the context of existing knowledge. | Introduction |
| Objectives | 4 | Provide an explicit statement of the objective(s) or question(s) the review addresses. | Introduction |
| **METHODS** | | |  |
| Eligibility criteria | 5 | Specify the inclusion and exclusion criteria for the review and how studies were grouped for the syntheses. | Methods  Supplement |
| Information sources | 6 | Specify all databases, registers, websites, organisations, reference lists and other sources searched or consulted to identify studies. Specify the date when each source was last searched or consulted. | Methods  Supplement |
| Search strategy | 7 | Present the full search strategies for all databases, registers, and websites, including any filters and limits used. | Methods  Supplement |
| Selection process | 8 | Specify the methods used to decide whether a study met the inclusion criteria of the review, including how many reviewers screened each record and each report retrieved, whether they worked independently, and if applicable, details of automation tools used in the process. | Methods  Supplement |
| Data collection process | 9 | Specify the methods used to collect data from reports, including how many reviewers collected data from each report, whether they worked independently, any processes for obtaining or confirming data from study investigators, and if applicable, details of automation tools used in the process. | Methods  Supplement |
| Data items | 10a | List and define all outcomes for which data were sought. Specify whether all results that were compatible with each outcome domain in each study were sought (e.g., for all measures, time points, analyses), and if not, the methods used to decide which results to collect. | Methods Supplement |
|  | 10b | List and define all other variables for which data were sought (e.g., participant and intervention characteristics, funding sources). Describe any assumptions made about any missing or unclear information. | Methods Supplement |
| Study risk of bias assessment | 11 | Specify the methods used to assess risk of bias in the included studies, including details of the tool(s) used, how many reviewers assessed each study and whether they worked independently, and if applicable, details of automation tools used in the process. | Methods Supplement |
| Effect measures | 12 | Specify for each outcome the effect measure(s) (e.g., risk ratio, mean difference) used in the synthesis or presentation of results. | Not applicable |
| Synthesis methods | 13a | Describe the processes used to decide which studies were eligible for each synthesis (e.g., tabulating the study intervention characteristics and comparing against the planned groups for each synthesis (item #5)). | Not applicable |
|  | 13b | Describe any methods required to prepare the data for presentation or synthesis, such as handling of missing summary statistics, or data conversions. | Not applicable |
|  | 13c | Describe any methods used to tabulate or visually display results of individual studies and syntheses. | Methods |
|  | 13d | Describe any methods used to synthesise results and provide a rationale for the choice(s). If meta-analysis was performed, describe the model(s), method(s) to identify the presence and extent of statistical heterogeneity, and software package(s) used. | Methods |
|  | 13e | Describe any methods used to explore possible causes of heterogeneity among study results (e.g., subgroup analysis, meta-regression). | Not applicable |
|  | 13f | Describe any sensitivity analyses conducted to assess robustness of the synthesised results. | Not applicable |
| Reporting bias assessment | 14 | Describe any methods used to assess risk of bias due to missing results in a synthesis (arising from reporting biases). | Methods |
| Certainty assessment | 15 | Describe any methods used to assess certainty (or confidence) in the body of evidence for an outcome. | Not applicable |
| **RESULTS** | | |  |
| Study selection | 16a | Describe the results of the search and selection process, from the number of records identified in the search to the number of studies included in the review, ideally using a flow diagram. | Figure 1 Results |
|  | 16b | Cite studies that might appear to meet the inclusion criteria, but which were excluded, and explain why they were excluded. | Supplement |
| Study characteristics | 17 | Cite each included study and present its characteristics. | Results  Table 1 |
| Risk of bias in studies | 18 | Present assessments of risk of bias for each included study. | Results Supplement |
| Results of individual studies | 19 | For all outcomes, present, for each study: (a) summary statistics for each group (where appropriate) and (b) an effect estimate and its precision (e.g., confidence/credible interval), ideally using structured tables or plots. | Table 1 |
| Results of syntheses | 20a | For each synthesis, briefly summarise the characteristics and risk of bias among contributing studies. | Results Supplement |
|  | 20b | Present results of all statistical syntheses conducted. If meta-analysis was done, present for each the summary estimate and its precision (e.g., confidence/credible interval) and measures of statistical heterogeneity. If comparing groups, describe the direction of the effect. | Not applicable |
|  | 20c | Present results of all investigations of possible causes of heterogeneity among study results. | Not applicable |
|  | 20d | Present results of all sensitivity analyses conducted to assess the robustness of the synthesised results. | Not applicable |
| Reporting biases | 21 | Present assessments of risk of bias due to missing results (arising from reporting biases) for each synthesis assessed. | Supplement |
| Certainty of evidence | 22 | Present assessments of certainty (or confidence) in the body of evidence for each outcome assessed. | Not applicable |
| **DISCUSSION** | | |  |
| Discussion | 23a | Provide a general interpretation of the results in the context of other evidence. | Discussion |
|  | 23b | Discuss any limitations of the evidence included in the review. | Discussion |
|  | 23c | Discuss any limitations of the review processes used. | Discussion |
|  | 23d | Discuss implications of the results for practice, policy, and future research. | Discussion |
| **OTHER INFORMATION** | | |  |
| Registration and protocol | 24a | Provide registration information for the review, including register name and registration number, or state that the review was not registered. | Discussion |
|  | 24b | Indicate where the review protocol can be accessed, or state that a protocol was not prepared. | Not applicable |
|  | 24c | Describe and explain any amendments to information provided at registration or in the protocol. | Not applicable |
| Support | 25 | Describe sources of financial or non-financial support for the review, and the role of the funders or sponsors in the review. | Funding |
| Competing interests | 26 | Declare any competing interests of review authors. | Competing interest declaration |
| Availability of data, code and other materials | 27 | Report which of the following are publicly available and where they can be found: template data collection forms; data extracted from included studies; data used for all analyses; analytic code; any other materials used in the review. | Article information |

#

# PRISMA 2020 for abstracts checklist

| **Section and Topic** | **Item #** | **Checklist item** | **Reported (Yes/No)** |
| --- | --- | --- | --- |
| **TITLE** | | |  |
| Title | 1 | Identify the report as a systematic review. | Yes |
| **BACKGROUND** | | |  |
| Objectives | 2 | Provide an explicit statement of the main objective(s) or question(s) the review addresses. | Yes |
| **METHODS** | | |  |
| Eligibility criteria | 3 | Specify the inclusion and exclusion criteria for the review. | Yes |
| Information sources | 4 | Specify the information sources (e.g. databases, registers) used to identify studies and the date when each was last searched. | Yes |
| Risk of bias | 5 | Specify the methods used to assess risk of bias in the included studies. | Yes |
| Synthesis of results | 6 | Specify the methods used to present and synthesise results. | Yes |
| **RESULTS** | | |  |
| Included studies | 7 | Give the total number of included studies and participants and summarise relevant characteristics of studies. | Yes |
| Synthesis of results | 8 | Present results for main outcomes, preferably indicating the number of included studies and participants for each. If meta-analysis was done, report the summary estimate and confidence/credible interval. If comparing groups, indicate the direction of the effect (i.e. which group is favoured). | Yes |
| **DISCUSSION** | | |  |
| Limitations of evidence | 9 | Provide a brief summary of the limitations of the evidence included in the review (e.g. study risk of bias, inconsistency and imprecision). | Yes |
| Interpretation | 10 | Provide a general interpretation of the results and important implications. | Yes |
| **OTHER** | | |  |
| Funding | 11 | Specify the primary source of funding for the review. | Yes |
| Registration | 12 | Provide the register name and registration number. | No |

# Risk of bias and quality assessment of included studies - Newcastle-Ottawa scale for non-randomised studies

The Newcastle-Ottawa Scale (NOS) (Wells et al., 2014) is a validated tool for non-randomised studies (cross-sectional and longitudinal), which assesses the quality of a study and the risk of bias. We can award a maximum of one point (star) for each numbered item with the Selection and Outcome/Exposure categories. A maximum of two points (stars) can be given for Comparability.

**Newcastle-Ottawa quality assessment scale cohort studies**

Note: A study can be awarded a maximum of one star for each numbered item within the Selection and Outcome categories. A maximum of two stars can be given for Comparability

***Selection***

1) Representativeness of the exposed cohort

1. truly representative of the average children, adolescents and adults in the community **🟑**
2. somewhat representative of the average children, adolescents and adults in the community **🟑**
3. selected group of users eg nurses, volunteers
4. no description of the derivation of the cohort

2) Selection of the non exposed cohort

1. drawn from the same community as the exposed cohort **🟑**
2. drawn from a different source
3. no description of the derivation of the non exposed cohort

3) Ascertainment of exposure

1. secure record **🟑**
2. structured interview **🟑**
3. written self report
4. no description

4) Demonstration that outcome of interest was not present at start of study

1. yes **🟑**
2. no

***Comparability***

1) Comparability of cohorts on the basis of the design or analysis

1. study controls for the absence of childhood trauma **🟑**
2. study controls for any additional factor **🟑**

***Outcome***

1) Assessment of outcome

1. independent blind assessment **🟑**
2. record linkage **🟑**
3. self report
4. no description

2) Was follow-up long enough for outcomes to occur

1. yes **🟑**
2. no

3) Adequacy of follow up of cohorts

1. complete follow up - all subjects accounted for **🟑**
2. subjects lost to follow up unlikely to introduce bias - small number lost - > 5 % **🟑**
3. follow up rate < 95 % and no description of those lost
4. no statement

**Newcastle-Ottawa quality assessment scale case control studies**

Note: A study can be awarded a maximum of one star for each numbered item within the Selection and Exposure categories. A maximum of two stars can be given for Comparability.

***Selection***

1) Is the case definition adequate?

1. yes, with independent validation **🟑**
2. yes, eg record linkage or based on self reports
3. no description

2) Representativeness of the cases

1. consecutive or obviously representative series of cases **🟑**
2. potential for selection biases or not stated

3) Selection of Controls

1. community controls **🟑**
2. hospital controls
3. no description

4) Definition of Controls

1. no history of disease **🟑**
2. no description of source

***Comparability***

1) Comparability of cases and controls on the basis of the design or analysis

1. study controls for the absence of childhood trauma **🟑**
2. study controls for any additional factor **🟑**

***Exposure***

1) Ascertainment of exposure

1. secure record **🟑**
2. structured interview where blind to case/control status **🟑**
3. interview not blinded to case/control status
4. written self report or medical record only
5. no description

2) Same method of ascertainment for cases and controls

1. yes **🟑**
2. no

3) Non-Response rate

1. same rate for both groups **🟑**
2. non respondents described
3. rate different and no designation

Four independent reviewers used the English version of the Newcastle-Ottawa Scale (NOS) for non-randomised studies to assess the quality of included studies; discrepancies were resolved through consensus among reviewers. Studies were assessed based on three broad domains: 1) Selection; 2) Comparability; 3) Outcome/Exposure.

The maximum number of scores for each domain was 4 for Selection, 2 for Comparability, and 3 for Outcome/Exposure. Total quality scores ranged from 0 to 9, with a higher score representing better quality. We rated the overall quality according to specific combinations of results across the three domains. The evaluations are shown below.

| **Study** | **Selection** | **Comparability** | **Outcome/Exposure** | **NOS stars** |
| --- | --- | --- | --- | --- |
| Alaftar & Uzer, 2022 | **** |  | * | 5 |
| Barry et al., 2021 | **** | ** | *** | 9 |
| Bendstrup et al., 2021 | ** | ** | *** | 7 |
| Berthelot et al., 2015 | ** | * | *** | 6 |
| Chiasson et al., 2022 | **** | ** | ** | 8 |
| Crane et al., 2014 | ** | ** | ** | 6 |
| D’Amico et al., 2022 | **** | * | ** | 7 |
| Dawson & Bryant, 2016 | ** | * | * | 4 |
| Ding & He, 2021 | *** | * | ** | 6 |
| Feurer et al., 2018 | **** | * | ** | 7 |
| Fishere & Habermas, 2023 | *** | ** | ** | 7 |
| Fohn et al., 2017 | * | * | * | 3 |
| Goldfarb et al., 2019 | ** | * | ** | 5 |
| Goldfarb et al., 2023 | *** | * | ** | 6 |
| Griffith et al., 2016 | *** | * | ** | 6 |
| Hakamata et al., 2021 | ** | * | ** | 5 |
| Harris et al., 2016 | ** | ** | *** | 7 |
| Hawkins et al., 2020 | *** | * | ** | 6 |
| Hitchcock et al., 2014 | *** |  | ** | 5 |
| Huntjens et al., 2014 | **** | ** | *** | 9 |
| Jiang et al., 2020 | **** | ** | *** | 9 |
| Kaczmarczyk et al., 2018 | **** | ** | *** | 9 |
| Kangaslampi, 2023 | *** |  | * | 4 |
| Kaynar & Er, 2015 | *** |  | ** | 5 |
| Lawson et al., 2021 | *** | * | ** | 6 |
| Lin et al., 2022 | *** | * | * | 5 |
| McCrory et al., 2017 | *** | * | ** | 6 |
| McKinnon et al., 2017 | ** | * | * | 4 |
| Neshat Doost et al., 2014 | *** | ** | *** | 8 |
| Pacheco & Scheeringa, 2022 | *** | ** | ** | 7 |
| Parlar et al., 2016 | *** | ** | *** | 8 |
| Peltonen et al., 2017 | **** | * | ** | 7 |
| Risløv Staugaard et al., 2017 | *** |  | ** | 5 |
| Saleh et al., 2017 | **** | ** | *** | 9 |
| Salomão et al., 2021 | *** | * | ** | 6 |
| Staniloiu et al., 2018 | ** |  | * | 3 |
| Thomson & Jaque, 2022 | **** | * | ** | 7 |
| Tian et al., 2018 | *** | ** | *** | 8 |
| Vallet et al., 2017 | *** | * | ** | 6 |
| Varnaseri et al., 2016 | ** |  | ** | 4 |
| Viard et al., 2019 | *** | ** | *** | 8 |
| Wang et al., 2016 | **** | * | ** | 7 |
| Weems et al., 2014 | **** | * | * | 6 |
| Wittekind et al., 2016 | *** | ** | *** | 8 |
| Wittekind et al., 2017 | **** | ** | *** | 9 |
| Wolf & Nochajski, 2022 | *** |  | ** | 5 |
| Zhang et al., 2023 | *** | * | * | 5 |
| Zhu & Hakim-Larson, 2022 | *** | * | * | 5 |

#

#

# References

Baker, D. E., Hill, M., Chamberlain, K., Hurd, L., Karlsson, M., Zielinski, M., et al. (2021). Interpersonal vs. Non-Interpersonal Cumulative Traumas and Psychiatric Symptoms in Treatment-Seeking Incarcerated Women. *Journal of Trauma & Dissociation* 22, 249–264. doi: 10.1080/15299732.2020.1760172.

Berntsen, D., and Rubin, D. C. eds. (2012). *Understanding Autobiographical Memory: Theories and Approaches*. 1st ed. Cambridge University Press doi: 10.1017/CBO9781139021937.

De Bellis, M. D., and Zisk, A. (2014). The Biological Effects of Childhood Trauma. *Child and Adolescent Psychiatric Clinics of North America* 23, 185–222. doi: 10.1016/j.chc.2014.01.002.

Maharaj, R., Tineo, K., Flores-Ortega, M., Cordova, D. A., Iskhakova, A., Linn, R., et al. (2022). The association between COVID-19-related stressors and mental health outcomes for survivors of past interpersonal and non-interpersonal trauma. *European Journal of Trauma & Dissociation* 6, 100300. doi: 10.1016/j.ejtd.2022.100300.

Musicaro, R. M., Ford, J., Suvak, M. K., Sposato, A., and Andersen, S. (2020). Sluggish cognitive tempo and exposure to interpersonal trauma in children. *Anxiety, Stress, & Coping* 33, 100–114. doi: 10.1080/10615806.2019.1695124.

Parker, G., Tupling, H., and Brown, L. B. (1979). A Parental Bonding Instrument. *British Journal of Medical Psychology* 52, 1–10. doi: 10.1111/j.2044-8341.1979.tb02487.x.

Teicher, M. H., and Samson, J. A. (2013). Childhood Maltreatment and Psychopathology: A Case for Ecophenotypic Variants as Clinically and Neurobiologically Distinct Subtypes. *AJP* 170, 1114–1133. doi: 10.1176/appi.ajp.2013.12070957.

Thomas, E. A., Owens, G. P., and Keller, E. M. (2021). Relationships among non‐interpersonal and interpersonal trauma types, posttraumatic stress, and posttraumatic growth. *J Clin Psychol* 77, 2592–2608. doi: 10.1002/jclp.23190.

Wells, G.A., Wells, G., Shea, B., Shea, B., O'Connell, D., Peterson, J., et al. (2014). *The Newcastle-Ottawa Scale (NOS) for Assessing the Quality of Nonrandomised Studies in Meta-Analyses*. <https://www.semanticscholar.org/paper/The-Newcastle-Ottawa-Scale-(NOS)-for-Assessing-the-Wells-Wells/c293fb316b6176154c3fdbb8340a107d9c8c82bf>
